# Spatial Transcriptomics Reveals Regional and Temporal Dynamics of Gene Expression in the Mouse Brain Across Development and Aging

**DOI:** 10.3390/biology14060717

**Published:** 2025-06-18

**Authors:** Benjamin Conacher, Amanda Moore, Liduo Yin, Yu Lin, Xiguang Xu, Qinwen Mao, Hehuang Xie

**Affiliations:** 1 Epigenomics and Computational Biology Lab, Fralin Life Sciences Institute, Virginia Tech, Blacksburg, VA 24061, USA; benconacher@vt.edu (B.C.); yinliduo@vt.edu (L.Y.); xiguang@vt.edu (X.X.); 2 Genetics, Bioinformatics and Computational Biology Program, Virginia Tech, Blacksburg, VA 24061, USA; 3 Department of Biomedical and Veterinary Sciences, Virginia-Maryland College of Veterinary Medicine, Virginia Tech, Blacksburg, VA 24061, USA; 4 Department of Pathology, Huntsman Cancer Institute, University of Utah, 2000 Circle of Hope Drive, Salt Lake City, UT 84112, USA; 5 Translational Biology, Medicine and Health Program, Virginia Tech, Blacksburg, VA 24061, USA; 6 School of Neuroscience, Virginia Tech, Blacksburg, VA 24061, USA

**Keywords:** spatial transcriptomics, brain development, aging, hippocampus, neuroinflammation, myelination, neurogenesis

## Abstract

Investigating transcriptomic changes during healthy development and aging provides insights into the molecular mechanisms that regulate the maturation of brain functions and drive age-related decline. Although it has been speculated that aging may represent a reversal of late-stage brain development, direct molecular comparisons between these two processes have remained limited. This study employs spatial transcriptomics to analyze the mouse brain at three key timepoints: postnatal day 21 (P21), 3 months (adult), and 28 months (aged), to identify region-specific differential gene expression dynamics. We identify widespread transcriptional changes across both brain development and aging, with all brain regions exhibiting distinct, region-specific gene expression dynamics that reflect divergent regulatory trajectories across the lifespan. During development, gene expression patterns were strongly enriched for neurogenesis, synaptic plasticity, and myelination, reflecting active circuit formation and white matter maturation. In contrast, aging was characterized by a decline in myelination-related gene expression and a pronounced increase in inflammatory and glial activation pathways, particularly within the hippocampus. While both development and aging involved changes in myelination-associated genes, the underlying mechanisms appear distinct: developmental upregulation supports circuit establishment and refinement, whereas aging-related downregulation may reflect secondary consequences of neuroinflammation and reactive gliosis. These findings underscore that, despite some overlap in affected pathways, neural maturation and age-related decline are driven by fundamentally different regulatory programs. These findings establish a novel spatial transcriptomic reference for brain development and aging, offering a valuable data resource for investigating neurodevelopmental and neurodegenerative mechanisms.

## Simple Summary

The brain changes dramatically throughout life, from childhood to old age. Understanding how different parts of the brain grow, mature, and decline is important for identifying the causes of developmental and age-related brain disorders. In this study, we examined the brains of mice at three life stages, early development, adulthood, and old age, using a spatial sequencing approach that maps where genes are actively expressed. We found that each brain region has a unique pattern of gene activity that changes over time. In younger brains, gene activity was focused on growth and brain development, including the formation of new brain cells and the insulation of nerve fibers. In contrast, aging brains showed increased activity in genes linked to inflammation and stress responses. We also found that several key genes known to support brain cells and influence the risk of neurodegeneration showed consistent changes in activity at different life stages. These findings help explain how healthy brain function is developed, maintained, or lost with age and may guide future strategies to prevent or treat developmental- or age-related brain disorders.

## 1. Introduction

The brain, one of the most dynamic organs in the body, undergoes substantial molecular, structural, and functional changes throughout the lifespan. These changes reflect the delicate balance between development, maintenance, and decline, orchestrated by transcriptomic and epigenetic mechanisms [1,2]. Early in life, rapid neurogenesis, synaptogenesis, and gliogenesis establish neural networks essential for cognitive development, sensory processing, and adaptive behaviors [3,4,5]. This developmental phase is marked by high plasticity, allowing the brain to respond to environmental stimuli and learning demands [6]. With aging, these processes shift toward maintaining homeostasis, repairing damage, and managing aging-related stressors [7]. This shift is characterized by reduced neurogenesis, diminished synaptic plasticity, and increased inflammatory and degenerative activity, ultimately affecting cognitive function and increasing disease susceptibility [8,9,10].

The hippocampus, a region critical for learning, memory, and spatial navigation, exemplifies these life-stage transitions [11]. In early development, the dentate gyrus within the hippocampus serves as a neurogenic hub, integrating new neurons into established circuits to support memory formation and adaptability [12]. However, during aging, the hippocampus undergoes significant alterations, including reduced synaptic density, decreased neurogenesis, and the accumulation of reactive astrocytes and microglia, contributing to a pro-inflammatory environment [13]. These changes underpin the hippocampus’s involvement in neurodevelopmental disorders like autism spectrum disorder and age-related conditions such as Alzheimer’s disease [14,15]. The hippocampus interacts dynamically with other brain regions, including the cortex, thalamus, and hypothalamus, forming networks critical for cognitive and emotional stability. However, these inter-regional connections deteriorate with aging [16,17]. Despite significant advancements in understanding brain development and aging, critical gaps remain in our knowledge of how transcriptomic changes are spatially and temporally regulated across the lifespan. Traditional transcriptomic approaches, such as bulk RNA sequencing, lack the spatial resolution necessary to capture region-specific molecular dynamics [1]. Recent advances in spatial transcriptomics, including the development of platforms like 10x Genomics Visium [18], now allow high-resolution mapping of gene expression within intact tissue architecture, providing new insights into age-related transcriptional shifts and region-specific vulnerability [19].

Recent studies have applied spatial aging clocks to predict cell-type-specific aging patterns. For instance, Sun et al. (2025) [19] developed spatially resolved single-cell transcriptomic atlases across the adult lifespan, revealing that T cells increasingly infiltrate the brain with age, exerting pro-aging effects on neighboring cells, while neural stem cells have rejuvenating proximity effects. Additionally, Wu et al. (2024) [20] generated spatial transcriptomic maps of young and old mouse brains, identifying region-specific aging-related gene expression changes, particularly in the isocortex and hippocampal formation. Despite these advancements, few studies have systematically compared spatial gene expression patterns across both neurodevelopmental and aging timepoints. Thus, the integration of spatial transcriptomics to investigate life-stage-dependent molecular transitions remains limited, and the mechanisms distinguishing neural maturation from age-related decline at the regional level are still poorly defined.

Here, we leverage spatial transcriptomics to analyze region-specific gene expression dynamics in the cortex, hippocampus, thalamus, hypothalamus, and striatum across early-life development and aging, providing a novel framework for understanding molecular transitions that shape brain function over time. This study examines the mouse brain at three key stages: postnatal day 21 (P21), representing a critical period of brain plasticity and developmental progression; adulthood (3 months), reflecting mature baseline function; and aging (28 months), a stage associated with cognitive decline and neurodegenerative vulnerability. By identifying spatially resolved molecular transitions and their potential regulators, our approach aims to uncover mechanisms underlying cognitive decline and refine our understanding of healthy brain development. These insights bridge gaps in our knowledge of spatial transcriptomic dynamics, offering a comparative perspective on how region-specific gene expression influences the hallmarks of development and aging.

## 2. Materials and Methods

### 2.1. Mouse Model

C57BL/6J mice were acquired from Jackson Laboratory (Bar Harbor, ME, USA) and maintained in a pathogen-free facility under a 12 h light/dark cycle. Male mice were harvested at postnatal day 21 (P21), 3 months (adult), and 28 months (aged). All animal procedures complied with national and international standards and were approved by the Virginia Tech Institutional Animal Care and Use Committee (IACUC).

### 2.2. FFPE Tissue Block Preparation

Mice were euthanized by CO2 inhalation followed by cervical dislocation. Brain tissue was promptly dissected, sectioned into 4 mm thick blocks, and fixed overnight at room temperature in 10% neutral buffered formalin (NBF). After fixation, tissues were placed in cassettes, gradually dehydrated through an ethanol series, and embedded in paraffin. The resulting formalin-fixed paraffin-embedded (FFPE) blocks were stored at 4 °C for subsequent analyses.

### 2.3. FFPE Visium Spatial Transcriptomics Library Preparation

The Visium Spatial Gene Expression (VGSE) libraries were constructed using Visium Spatial Gene Expression v1 (10x Genomics, Pleasanton, CA, USA; cat# PN-1000185) for P21 and adult samples and Visium Spatial Gene Expression v2 (10x Genomics, cat# PN-1000863) for aged samples, following the manufacturer’s User Guides (CG000407 for v1; CG000543 for v2). Each Visium slide contains circular capture spots with a diameter of approximately 55 μm, each typically capturing mRNA transcripts from 1 to 10 cells depending on local tissue density.

For P21 and adult samples, FFPE tissue blocks were sectioned at a 5 μm thickness using a HistoCore MultiCut microtome (Leica Microsystems, Buffalo Grove, IL, USA; cat# 149Multi0C1). Sections were placed in a 39 °C water bath for 1 min, then directly mounted onto Visium v1 Spatial Gene Expression Slides. The slides were incubated on a thermal cycler at 42 °C for 3 h and stored at room temperature in a desiccator overnight. After a 60 °C incubation for 2 h, deparaffinization was performed with xylene and graded ethanol. The sections were H&E stained, imaged at 10× magnification on a MoticEasy Slide scanner (Motic, Richmond, BC, Canada), and saved as TIFF files. Following imaging, tissue decrosslinking, permeabilization, and hybridization with mouse WT probes were performed. Library preparation steps included probe ligation, washing, RNA digestion, probe release, probe extension, and probe elution. qPCR was performed to determine optimal PCR cycles, and SPRIselect beads were used for PCR purification. Library quality was checked using a Qubit 3.0 Fluorometer (Thermo Fisher Scientific, Waltham, MA, USA; cat# Q33216) and the 4150 TapeStation System (Agilent Technologies, Santa Clara, CA, USA; cat# G2992AA The libraries were pooled and sequenced by Novogene Co., Ltd. (Sacramento, CA, USA) on a NovaSeq 6000 System (Illumina, San Diego, CA, USA) at an approximate depth of 300 million read pairs per sample.

For aged samples, FFPE tissue blocks were sectioned at 5 μm thickness and initially placed onto standard glass slides. The slides underwent H&E staining, brightfield imaging, and transcriptomic probe hybridization, following a similar workflow as the P21 and adult samples. Library preparation followed Visium v2 protocol modifications, with enhanced probe design for increased transcript capture sensitivity. Probe ligation, RNA digestion, and tissue removal were carried out using the CytAssist instrument (10x Genomics, Pleasanton, CA, USA; cat# PN-1000444) in coordination with the transfer of tissue sections onto Visium v2 Spatial Gene Expression Slides. Probe extension, elution, amplification, library construction, library quality control, and sequencing followed a similar workflow as the P21 and adult samples described above.

Sequencing reads for all samples underwent the following protocol: read 1: 151 cycles, i7 index read: 10 cycles, i5 index read: 10 cycles, and read 2: 151 cycles.

### 2.4. Analysis of FFPE Visium Spatial Transcriptomics Data

The FFPE Visium sequencing reads were aligned to the mouse reference genome (mm10) using 10x Genomics Space Ranger v2.0.0 for P21 and adult samples and Space Ranger v3.1.1 for aged samples, following the manufacturer’s recommendations. Alignment and gene expression quantification were performed using the short-read probe alignment algorithm for FFPE through the ‘count’ method. The resulting gene count matrices were then utilized in downstream analysis with the Seurat package (v5.1.0) in R [21]. All resulting gene count matrices were combined into a single Seurat object for comprehensive processing and analysis. This pooled object, containing all six biological replicates (two per age group), was used for all downstream normalization, dimensionality reduction, batch correction with Harmony, UMAP visualization, and clustering. The dataset was refined by (1) discarding spots containing fewer than 250 unique genes, and (2) removing spots with fewer than 500 unique molecular identifiers (UMIs). Across all hippocampal subregions, the number of spatial transcriptomic spots contributing to differential expression analysis ranged from 30 to 252, providing a sufficient resolution and statistical power for downstream comparisons. Detailed spot counts per hippocampal subregion and sample are provided in Supplementary Table S1.

Normalization was performed using the counts per million (CPM) method, followed by scaling to standardize expression values across all spatial spots. Dimensionality reduction was conducted via Principal Component Analysis (PCA), selecting the top 10 principal components for downstream analysis. Batch effects were mitigated using Harmony [22]. Uniform manifold approximation and projection (UMAP) was performed to visualize spatial transcriptomic patterns. The 3000 most variable genes were used for spatial clustering via the shared nearest neighbors (SNN) approach (‘FindNeighbors’ function). The ‘FindClusters’ function determined an optimal resolution of 0.075, generating biologically relevant clusters. These clusters were manually annotated based on tissue histology and the expression of known cell-type and brain-region marker genes using the ‘CellSelector’ function. In this study, DEGs were identified for each cluster using the ‘FindMarkers’ function with a pairwise Wilcoxon Rank Sum test [21]. DEGs were considered significant if they had an adjusted *p*-value < 0.05 and a fold change > 1.5, were expressed in >10% of spots within the cluster of interest, and were also detected in at least 10% of all spots across all samples. GO enrichment analysis was performed using the ‘clusterProfiler’ package (v4.6.2) in R [23] to identify biological processes associated with the significant DEGs. Figures 2e,f and 3d,e display heatmaps of enriched GO terms for up- and downregulated genes; however, the specific genes contributing to each GO term are provided in Supplementary Tables S2 and S3. To evaluate clustering robustness, silhouette scores were calculated based on the first 10 principal components using the cluster R package (v2.1.8.1) [24]. These scores confirmed strong intra-cluster cohesion and inter-cluster separation across most regions, with the results shown in Supplementary Figure S1a.

## 3. Results

### 3.1. FFPE Visium Spatial Transcriptomics Recaptures the Major Brain Regions

**Figure 1:**
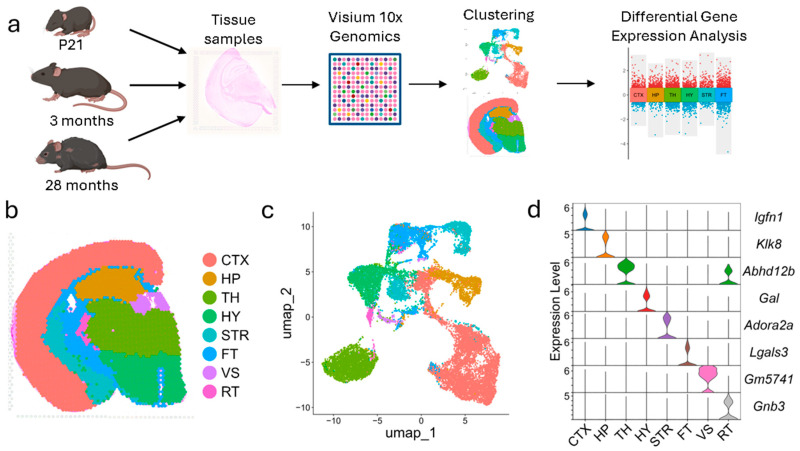
Experimental design and spatial clustering of mouse brain transcriptomes. (**a**) Schematic overview of the experimental design. Mice at P21, 3 months, and 28 months of age were harvested for spatial transcriptomics analysis to assess developmental and aging-associated changes in brain gene expression. (**b**) Representative feature plot showing spatial clusters corresponding to major anatomical regions: Cortex (CTX), Hippocampus (HP), Thalamus (TH), Hypothalamus (HY), Striatum (STR), Fiber tracts (FT), Ventricular system (VS), and Reticular nucleus of the thalamus (RT). (**c**) UMAP visualization of clusters by transcriptomic profiles. (**d**) Violin plot of cluster marker genes by region.

We applied the Visium spatial transcriptomics technique to investigate gene expression across brain regions in mice at three developmental stages: postnatal day 21 (P21), adult (3 months), and aged (28 months) (Figure 1a). Spatial transcriptomics was performed using the 10x Genomics Visium-FFPE platform, with two biological replicates per group, as described in the Methods. Each Visium slide captured between 2917 and 3679 spatial spots, with median counts of 6085 genes and 17,459 UMIs per spot, ensuring robust transcriptomic coverage (Supplementary Figure S1b–d).

Gene-count matrices were normalized using the counts per million (CPM) method to account for sequencing depth variability across samples (Supplementary Figure S2a,b). Subsequent clustering analysis identified eight distinct regions corresponding to major anatomical structures of the mouse brain: the cortex (CTX), hippocampus (HP), thalamus (TH), hypothalamus (HY), striatum (STR), fiber tracts (FT), ventricular system (VS), and reticular thalamus (RT) (Figure 1b,c, Supplementary Figure S2c). The anatomical identities of these clusters were established by comparing their spatial boundaries to reference images from the Allen Institute Mouse Brain Reference Atlas. This comparison demonstrated a high degree of alignment, confirming that the clustering analysis recapitulates the primary anatomical regions of the mouse brain. These spatial clustering patterns were consistent across biological replicates, and transcriptomic profiles demonstrated high inter-replicate correlation by brain region (Supplementary Figures S2c, S3 and S4a), further supporting the reproducibility and robustness of the dataset. To further validate the anatomical specificity of the annotated regions, we analyzed region-specific gene expression markers. Marker genes unique to each cluster were identified and shown to exhibit distinct expression patterns within their respective brain regions (Figure 1d). These findings confirm the validity and anatomical specificity of the transcriptomic data.

Overall, these results demonstrate that the FFPE Visium platform reliably identifies region-specific transcriptomic signatures across developmental stages. This consistency and robustness of these findings establish its utility for investigating the spatial organization of gene expression and the molecular basis of regional brain function across development and aging.

### 3.2. Brain Region-Specific Gene Expression Changes Across Developmental Stages

**Figure 2:**
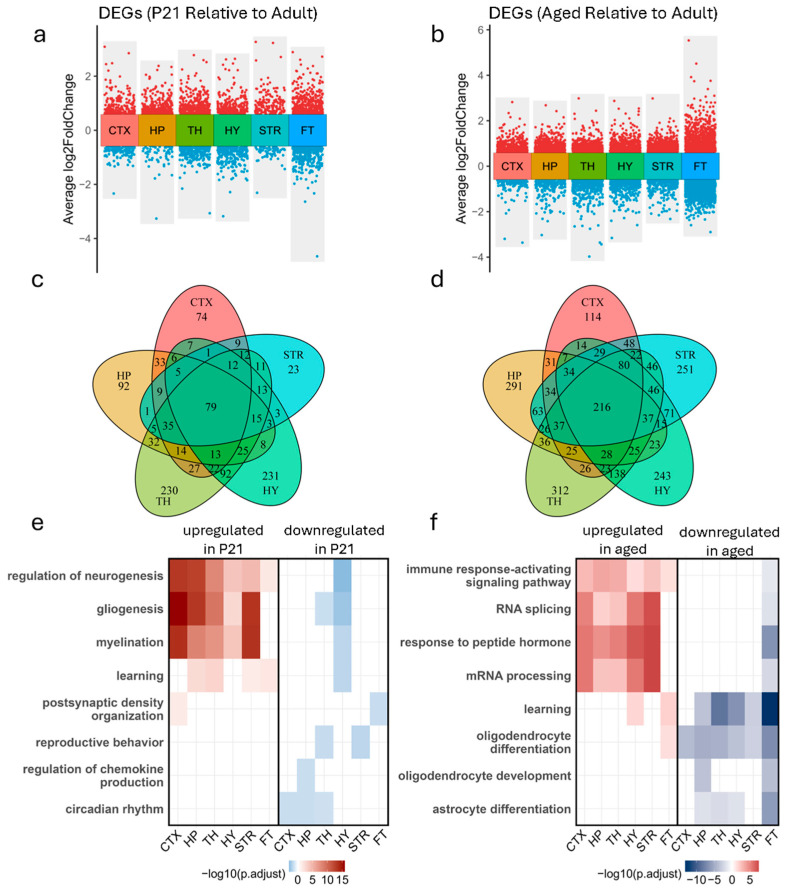
Region-specific differential gene expression across developmental and aging stages. Positive log2FC values indicate genes upregulated in P21 (a/e) or Aged (b/f) relative to Adult. (**a**) DEGs in P21 vs. adult samples by brain region. (**b**) DEGs in aged vs. adult samples by brain region. (**c**) Venn diagram of DEGs by brain region in P21 vs. adult samples. (**d**) Venn diagram of DEGs by brain region in aged vs. adult samples. (**e**) Heatmap of top two enriched biological functions per brain region in P21 vs. adult samples. (**f**) Heatmap of top two enriched biological functions per brain region in aged vs. adult samples.

We conducted differential gene expression analysis for each brain region, comparing P21 versus adult and aged versus adult samples (Figure 2a,b). Due to insufficient spot representation for reliable transcriptomic analysis, the regions corresponding to the ventricular system (VS) and reticular thalamus (RT) were excluded from downstream analyses. In the comparison between P21 and adult brains, it showed region-specific differentially expressed genes (DEGs), including 74 cortex-specific, 92 hippocampus-specific, 23 striatum-specific, 230 thalamus-specific, and 231 hypothalamus-specific DEGs. Moreover, 79 DEGs were shared by the five brain regions (Figure 2c). Interestingly, more region-specific DEGs were identified in the comparison between aged and adult brains. It showed 114 cortex-specific, 291 hippocampus-specific, 251 striatum-specific, 312 thalamus-specific, and 243 hypothalamus-specific DEGs. Additionally, 216 DEGs were shared among the five brain regions in the aged vs. adult comparison (Figure 2d). These results indicate that, during development or aging processes, both shared and region-specific gene expression changes were present across brain regions.

Gene Ontology (GO) enrichment analysis of DEGs in P21 mice relative to adult brains revealed robust region-specific activation of developmental programs across all major brain regions. Upregulated genes in P21 samples were consistently enriched in biological processes associated with neurogenesis, gliogenesis, myelination, and learning (Figure 2e). These terms were shared across CTX, HP, TH, HY, and STR, underscoring a widespread transcriptional emphasis on neural circuit formation and glial support. Key developmental markers such as *Plp1*, *Sox10*, and *Mbp* were prominently expressed in regions undergoing active myelination and glial differentiation in P21 mice (Supplementary Figure S5a). In contrast, genes downregulated in P21 mice relative to adults displayed diverse region-specific signatures. Enriched terms included postsynaptic density organization in the hippocampus, reproductive behavior in the hypothalamus, regulation of chemokine production in the striatum, and circadian rhythm processes, indicating a coordinated downregulation of adult-like functional states as the brain transitioned into maturity.

In aged mice compared to adult mice, a markedly different transcriptional profile emerged, characterized by increased expression of genes linked to inflammation and cellular stress responses. Across all but FT brain regions, genes upregulated in aged samples were enriched for immune response-activating signaling pathways, RNA splicing, response to peptide hormones, and mRNA processing (Figure 2f), reflecting a conserved aging-related activation of immune surveillance and transcriptional regulation machinery. Notably, genes such as *Apoe*, *Trem2*, and *Vegfa* were consistently elevated in aged brains (Supplementary Figure S5b), indicating widespread neuroinflammatory and angiogenic signaling. These findings align with recent studies demonstrating that RNA processing and alternative splicing are significantly altered with age. A meta-analysis across 11 animal species, including mice, found that aging is associated with increased intron retention and the accumulation of premature termination codon-containing transcripts, particularly in brain tissue, suggesting widespread dysregulation of RNA maturation as a conserved aging hallmark [25]. Complementing this, Kumar et al. (2024) reported widespread changes in transcript isoform usage in the aged mouse brain, including a decline in mRNA isoforms subjected to nonsense-mediated decay and altered binding of RNA-binding proteins, indicating a complex remodeling of post-transcriptional regulatory networks during aging [26].

Conversely, genes downregulated in aged samples revealed a progressive decline in biological processes critical for maintaining neural integrity. These included learning, oligodendrocyte differentiation, oligodendrocyte development, and astrocyte differentiation—processes critical for synaptic plasticity and white matter integrity. Notably, oligodendrocyte differentiation was consistently downregulated across all brain regions in aged mice, highlighting a global decline in myelination capacity with age. In contrast, learning-associated pathways were suppressed in all regions except the cortex, suggesting relative preservation of cognitive-associated transcriptional programs in this area. Downregulation of oligodendrocyte development was specific to the hippocampus and fiber tracts, while astrocyte differentiation was reduced in the hippocampus, thalamus, hypothalamus, and fiber tracts, reflecting regionally targeted glial dysfunction during aging.

### 3.3. Hippocampus Exhibits Subregion-Specific Transcriptomic Changes Across Development and Aging

**Figure 3:**
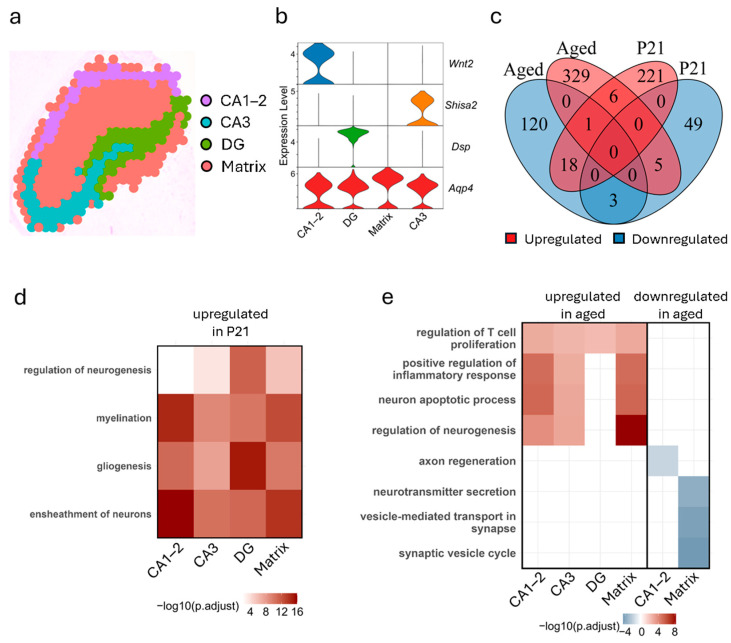
Subclustered analysis of hippocampal spatial transcriptomic profiles. (**a**) Representative spatial distribution of hippocampal subclusters. (**b**) Expression profiles of marker genes across subclusters. (**c**) Overlap of shared DEGs (upregulated/downregulated) between aged and P21 samples. (**d**) Gene ontology (GO) term enrichment analysis for significantly upregulated DEGs in P21 vs. adult samples. (**e**) GO term enrichment analysis for significantly up- and downregulated DEGs in aged vs. adult samples.

To explore age-dependent transcriptional changes in the hippocampus, we performed subclustering to delineate four distinct subregions: dentate gyrus (DG), Cornu Ammonis (CA) subfields CA1–2 and CA3, and the matrix (Figure 3a, Supplementary Figure S4a). Subregional marker genes, such as *Wnt2* (CA1–2), *Shisa2* (CA3), and *Dsp* (DG), and *Aqp4* (Matrix) confirmed the unique transcriptional profiles of these regions (Figure 3b). Comparative analysis of DEGs in P21 vs. adult and aged vs. adult hippocampal samples identified a small set of 32 shared genes, underscoring the largely distinct transcriptional landscapes that characterize early development and aging (Figure 3c).

To further investigate this phenomenon, we examined DEGs that were downregulated in P21 and upregulated in aged mice (“trending up”) or upregulated in P21 and downregulated in aged mice (“trending down”) (Supplementary Figure S4b), revealing spatially distinct patterns within hippocampal subregions. In the DG, *Lct* and *Ighm* were trending up, suggesting late-life activation following developmental repression. In the matrix subregion, *Ptgds* also followed a trending-up pattern. Conversely, genes exhibiting trending-down dynamics included *Tspan2* and *Fxyd7* in CA1–2, *Opalin* and *Tmem163* in DG, *Ttr*, *Tmem163*, *Hbb-bs*, and *Hba-a2* in CA3, and *Tspan2*, *Tmem163*, and *Fa2h* in the matrix. Notably, *Tmem163* appeared in multiple subregions, suggesting a shared transcriptional trajectory across distinct hippocampal compartments.

GO enrichment analysis revealed distinct spatial and temporal patterns of gene regulation across hippocampal subregions during development and aging. In the P21 vs. adult comparison, upregulated DEGs were consistently enriched for processes associated with myelination, gliogenesis, and ensheathment of neurons across all subregions, reflecting ongoing maturation of glial and structural support systems (Figure 3d). However, the degree of enrichment varied substantially across hippocampal subregions. For instance, regulation of neurogenesis showed the strongest enrichment in the DG and the weakest in CA1–2. This is consistent with the known biology of hippocampal development, in which the DG serves as a key site of postnatal neurogenesis, while the CA1–2 and CA3 regions comprise pyramidal neurons that are generated predominantly during embryogenesis and display minimal neurogenic activity after birth [12]. In contrast, CA1–2 exhibited the highest enrichment for myelination and neuron ensheathment, suggesting earlier maturation of its pyramidal neuron population. Gliogenesis was most enriched in the DG, with moderate levels observed in CA1–2 and the matrix. These findings indicate that while core developmental programs are activated across the hippocampus during early postnatal life, the magnitude of transcriptional engagement varies in a region-specific manner, likely reflecting differences in cell-type composition, developmental timing, and functional specialization.

In contrast, the aged vs. adult comparison showed an enrichment of immune and stress-related pathways among upregulated DEGs, including regulation of T-cell proliferation in all subregions, as well as positive regulation of the inflammatory response, neuron apoptotic process, and neurogenesis in all subregions except DG (Figure 3e). This lack of transcriptional response in the DG is consistent with prior observations that the DG maintains relatively stable levels of adult hippocampal neurogenesis during healthy aging, with significant declines reported primarily in the context of neurodegenerative disease such as Alzheimer’s [27]. This muted response may reflect lower transcriptional plasticity or a reduced inflammatory and apoptotic signaling burden in this region under normal aging conditions. In contrast, regulation of neurogenesis, inflammation, and apoptosis showed progressively higher enrichment in CA3, CA1–2, and especially the matrix region. While the matrix is not classically considered a neurogenic zone, its heightened transcriptional reactivity in aged samples may reflect compensatory remodeling, gliosis, or increased vulnerability to aging-related cellular stress. Notably, this suggests that while DG retains neurogenic potential with age, adjacent hippocampal subregions may undergo greater transcriptional shifts in response to aging-related insults.

Downregulated DEGs in the aged hippocampus were associated with reduced axon regeneration in CA1–2 and a diminished neurotransmitter secretion, vesicle-mediated synaptic transport, and synaptic vesicle cycle in the matrix, while no significant downregulated GO terms were identified in CA3 or DG. Supporting these trends, Venn diagram analysis (Supplementary Figure S6) showed that the total number of downregulated DEGs was very low in the P21 vs. adults, and modest in the aged vs. adults, whereas upregulated DEGs were more abundant in both comparisons, with the majority of genes being unique to individual subregions, highlighting the transcriptional heterogeneity of the hippocampus across the lifespan.

### 3.4. Differential Gene Expression Trends Across Life Stages

**Figure 4:**
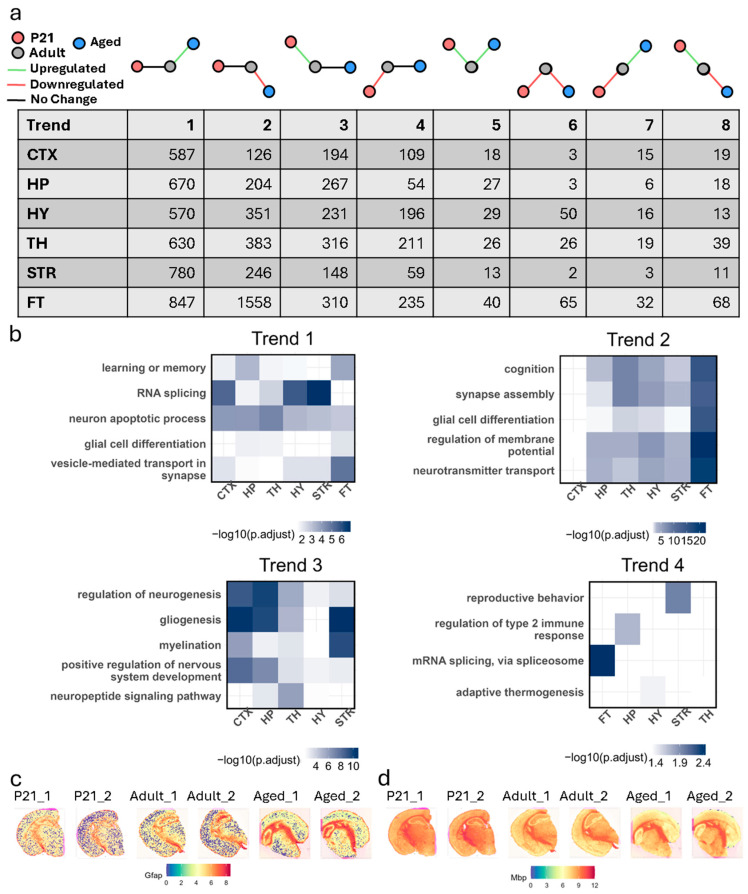
Trends in gene expression changes from development to aging across brain regions. (**a**) Number of DEGs categorized by trend type across brain regions. (**b**) Heatmap of hippocampal DEGs organized by trend type, alongside top enriched GO terms. (**c**) Spatial feature plot showing expression of Gfap, trending upward with aging. (**d**) Spatial feature plot showing expression of Mbp, trending downward with aging.

To elucidate transcriptional dynamics across life stages, we analyzed eight distinct trends of gene expression changes in major brain regions—cortex, hippocampus, hypothalamus, striatum, and thalamus—comparing P21 and aged samples against adult controls. These trends included genes upregulated in aged samples (trend 1), downregulated in aged (trend 2), upregulated in P21 (trend 3), downregulated in P21 (trend 4), upregulated in both P21 and aged (trend 5, up/up), downregulated in both (trend 6, down/down), downregulated in P21 but upregulated in aged s (trend 7, down/up), and upregulated in P21 but downregulated in aged samples (trend 8, up/down) (Figure 4a). To characterize the transcriptional programs underpinning neural development and aging, we conducted GO enrichment analysis for trends 1–4 across the cortex, hippocampus, and thalamus for regions and trends with at least 100 DEGs (Figure 4b).

#### 3.4.1. Trend 1: Upregulated in Only Aged Samples

Genes uniquely upregulated in aged samples were enriched for neuron apoptotic processes across all regions, suggesting a widespread shift in cell survival signaling with age. RNA splicing was enriched in most regions except fiber tracts, reflecting possible region-dependent changes in post-transcriptional regulation. Enrichment of vesicle-mediated transport at synapses and glial cell differentiation in the hippocampus, thalamus, and fiber tracts points to alterations in synaptic remodeling and glial activity in aging tissue. Additionally, the enrichment of learning or memory terms in most regions, excluding the striatum, may indicate transcriptional compensation or plasticity, though functional consequences remain to be determined.

#### 3.4.2. Trend 2: Downregulated in Only Aged Samples

Genes downregulated exclusively in aged samples were predominantly enriched for processes essential to synaptic integrity and excitability, including regulation of membrane potential, and synapse assembly, neurotransmitter transport, glial cell differentiation, and cognition in all but the cortex. The absence of these terms in cortical regions suggests differential vulnerability or transcriptional resilience in this area relative to subcortical structures. Together, these findings support a regionally heterogeneous decline in neuronal and glial transcriptional programs in the aged brain.

#### 3.4.3. Trend 3: Upregulated in Only P21 Samples

Genes specifically upregulated during postnatal development showed strong enrichment for well-characterized neurodevelopmental processes, including neurogenesis, myelination, and positive regulation of nervous system development across all regions. Enrichment of gliogenesis was detected in most regions except the hypothalamus, and terms related to neuropeptide signaling were present in all but the cortex and striatum. These findings are consistent with coordinated transcriptional activity supporting structural maturation, glial lineage progression, and early circuit establishment during brain development.

#### 3.4.4. Trend 4: Downregulated in Only P21 Samples

This trend included a small set of region-specific enrichment terms, such as regulation of the type 2 immune response (hippocampus), reproductive behavior (striatum), mRNA splicing via the spliceosome (fiber tracts), and adaptive thermogenesis (hypothalamus, thalamus). These annotations may reflect the suppression of non-neurodevelopmental transcriptional programs during this early stage, though the biological interpretation of these region-specific findings is limited by the broad nature of the terms and the absence of consistent enrichment across brain regions.

#### 3.4.5. Trend 5: Upregulated in Both Comparisons (Up/Up)

Genes upregulated in both P21 and aged samples were enriched for pathways linked to neurogenesis, synaptic organization, and glial differentiation, highlighting their roles in sustaining neural plasticity across life stages. In the hippocampus, *Nrp1* was associated with neuron projection extension, while cortical genes such as *Ctnnb1* and *Cdkn1a* contributed to synaptic maintenance and cell cycle regulation. Subregion-specific analysis revealed that the DG is a key contributor to neurogenesis, emphasizing its role in preserving functional connectivity throughout life.

#### 3.4.6. Trend 6: Downregulated in Both Comparisons (Down/Down)

Genes downregulated in both P21 and aged samples were enriched for pathways related to oxidative stress, protein trafficking, and neuroimmune interactions. For example, *Qdpr* and *Sec11c* were downregulated in the hippocampus, reflecting a conserved decline in cellular maintenance functions. The cortex showed a reduced expression of *Il33*, a neuroimmune modulator, suggesting systemic vulnerabilities in cellular resilience that may contribute to cognitive decline.

#### 3.4.7. Trend 7: Downregulated in P21 but Upregulated in Aged Samples (Down/Up)

This trend captured a shift from developmental suppression to aging-related activation of immune and glial remodeling pathways. In the hippocampus, genes such as *C4b* and the astrocytic marker *Gfap* were upregulated in aged samples (Supplementary Figure S7), reflecting gliosis and neuroinflammation. Similarly, in the cortex, stress-response genes like *Bhlhe40* and *Hspa1b* were activated in aged samples, potentially indicating cortical adaptive mechanisms. Spatial sequencing highlighted CA1–2 regions as particularly vulnerable, with reduced synaptic organization genes (*Gap43*, *Ntng2*), indicating synaptic decline during aging.

#### 3.4.8. Trend 8: Upregulated in P21 but Downregulated in Aged Samples (Up/Down)

Genes in this trend were associated with myelination and axon ensheathment, reflecting gliogenesis during development that diminishes with age. Myelin-related genes such as *Plp1*, *Cnp*, and *Mbp* were upregulated in P21 samples across all regions but were suppressed in aged samples (Supplementary Figure S8). This decline in myelination processes may contribute to reduced neural signal efficiency and cognitive function during aging.

## 4. Discussion

This study provides a spatially resolved transcriptomic analysis of gene expression changes across key life stages, revealing distinct molecular transitions from early development to aging. By leveraging high-resolution spatial transcriptomics, we identified regional and temporal shifts in gene expression that underpin the structural and functional changes in the aging brain. Importantly, this study aligns with prior work on the spatial organization of brain transcriptomes and highlights both shared and unique gene expression patterns associated with neural senescence [16,19,20,28,29,30].

Our analysis reveals two predominant transcriptional trends that characterize the transition from development to aging: a shift from developmental suppression to aging-related activation of immune and glial remodeling pathways, and a decline in myelination-associated processes. Specifically, we observed upregulation of *C4b* and *Gfap* in the aged hippocampus, consistent with gliosis and neuroinflammation, and activation of stress-response genes (*Bhlhe40*, *Hspa1b*) in the cortex, potentially reflecting adaptive mechanisms to cellular stress. In parallel, genes associated with myelination and axon ensheathment (*Plp1*, *Cnp*, *Mbp*), which were robustly expressed in P21 samples, were downregulated in aged brains, suggesting a progressive decline in myelin integrity that may contribute to cognitive deterioration.

Our findings align with prior studies reporting myelination deficits and inflammation during aging [31,32] but offer critical new insights into the spatial specificity of these changes. While Sun et al. (2025) [19] reported an increase in oligodendrocyte proportions in aging white matter tracts, they also found a concurrent decline in OPCs in these regions, suggesting reduced oligodendrocyte renewal capacity. Our findings build on this by demonstrating that, despite an increase in oligodendrocytes, key myelination genes (*Plp1*, *Cnp*, *Mbp*) are downregulated in aged samples, pointing to an impairment in myelin maintenance rather than sustained oligodendrocyte function. This discrepancy aligns with studies showing that chronic neuroinflammation disrupts oligodendrocyte maturation and remyelination [33,34], reinforcing the idea that inflammatory signaling, rather than oligodendrocyte depletion per se, is a primary driver of myelin degeneration in aging.

In addition to these broad developmental and aging transitions, our analysis of genes showing opposing regulation across life stages (“trending up” or “trending down”) uncovered subregion-specific, biphasic expression patterns within the hippocampus. For example, *Lct*, *Ighm*, and *Ptgds* were repressed during development but activated in aging, suggesting late-life roles in stress response or immune modulation. Conversely, genes such as *Tspan2*, *Tmem163*, and *Fa2h*, which were robustly expressed during development, were downregulated with age in CA1–2, DG, CA3, and matrix subregions, implicating their loss in region-specific decline of synaptic, myelin-related, or metabolic functions. The repeated appearance of *Tmem163* across multiple subregions highlights its potential as a key mediator of hippocampal remodeling over time. These findings support a model in which a select set of genes is dynamically regulated in a spatially distinct manner to facilitate either circuit maturation during development or compensatory adaptation in aging. Additionally, while previous studies have broadly characterized transcriptional changes in aging, our spatial transcriptomic approach provides a more refined anatomical framework that maps these changes to specific hippocampal subregions. Rather than uniform vulnerability, we observed region-specific transcriptional trajectories, with distinct sets of genes altered in CA1–2, CA3, DG, and the matrix. This spatial heterogeneity underscores the importance of capturing local gene expression dynamics to better understand the diverse molecular processes contributing to hippocampal aging and to identify subregion-specific targets for therapeutic intervention.

The upregulation of *C4b* and *Gfap* in aged hippocampal samples suggests that modulating astrocyte reactivity and complement activation may be a strategy to mitigate age-related synaptic damage. Likewise, the upregulation of *Trem2* and *Apoe*, key regulators of microglial activation, suggests that TREM2-targeted therapies, which have shown promise in neurodegenerative disease models [35], may hold therapeutic potential for mitigating neuroinflammatory damage in aging. In parallel, the downregulation of *Plp1*, *Cnp*, and *Mbp* suggests that enhancing oligodendrocyte function may restore myelin integrity. Agents such as metformin have demonstrated efficacy in promoting remyelination by rejuvenating aged OPCs, thereby serving as potential therapeutic candidates for preserving myelin function in aging populations [36]. Additionally, fractalkine has shown promise in stimulating oligodendrocyte production and remyelination, offering another avenue for therapeutic intervention [37].

Despite the insights gained from this spatially resolved analysis, several limitations warrant consideration. First, the 10x Genomics Visium platform operates at a resolution of ~55 µm, capturing transcriptomes from multiple cells within each spot. While regional trends were robust, conclusions regarding cell-type-specific processes, such as astrocyte activation or oligodendrocyte maturation, should be interpreted with caution. Future studies that integrate single-cell transcriptomic or epigenomic datasets with spatial profiles will be essential to definitively resolve the cellular contributors to aging-associated gene expression changes. Second, this study captures only three discrete life stages (P21, 3 months, and 28 months), leaving a substantial temporal gap between adulthood and aging. As such, the data cannot fully characterize the continuity or rate of molecular changes across the lifespan. Inclusion of intermediate timepoints will help define more precise aging trajectories. Finally, incorporation of complementary multi-omic layers, such as proteomics, metabolomics, or spatially resolved chromatin accessibility, may offer deeper mechanistic insight into how transcriptional changes influence cellular function and vulnerability during brain aging.

## 5. Conclusions

This study provides a comprehensive spatial map of gene expression changes in the mouse brain across development and aging, highlighting region-specific and life-stage-dependent transcriptional programs. By comparing early postnatal, adult, and aged brains, we identified distinct molecular signatures associated with neurogenesis, myelination, and glial activation that vary across brain regions and over time. Developmental stages were marked by widespread activation of genes involved in neural circuit formation, while aging was characterized by increased expression of inflammatory and stress-related genes and a decline in myelination-associated pathways. These findings underscore the complexity of brain aging and demonstrate that age-related decline is not simply a reversal of development but involves unique molecular processes. Our dataset offers a valuable resource for understanding brain maturation and vulnerability and may inform future research into mechanisms of cognitive decline and neurodegeneration.

## Data Availability

The Visium spatial transcriptomics datasets generated and analyzed in this study have been deposited in the NCBI Gene Expression Omnibus (GEO) under accession number GSE287202. All original code used for data processing and analysis is available on GitHub at https://github.com/bconacher/spatial-transcriptomics-mouse-aging (accessed on 7 May 2025). Additional information required to reanalyze the data is available from the corresponding author upon reasonable request.

## Supplementary Materials

The following supporting information can be downloaded at https://www.mdpi.com/article/10.3390/biology14060717/s1, Figure S1: Quality Control Metrics and Clustering Robustness of Spatial Transcriptomics Data; Figure S2: Replicate Correlation and Housekeeping Gene Normalization; Figure S3: Gene Expression Correlation Between Biological Replicates; Figure S4: Subregion-Specific Spatial Clustering and Differential Gene Expression in the Hippocampus; Figure S5: Representative Expression Patterns of Myelination- and Inflammation-Associated Genes; Figure S6: Venn Diagrams of DEGs by Sample and Hippocampal Subcluster; Figure S7: Heatmaps of Top Genes Trending Up by Region; Figure S8: Heatmaps of Top Genes Trending Down by Region; Table S1: Spatial Transcriptomic Spot Counts per Sample and Hippocampal Subregion; Table S2: Genes Associated with Enriched GO Terms for P21 DEGs; Table S3: Genes Associated with Enriched GO Terms for Aged DEGs.
