# Supplementary material for: Spatial Transcriptomics Reveals Regional and Temporal Dynamics of Gene Expression in the Mouse Brain Across Development and Aging
Source: Biology (Basel). 2025 Jun 18;14(6):717. doi: 10.3390/biology14060717 (PMC12189371; doi:10.3390/biology14060717)
Supplement: Supplementary file 1 [file biology-14-00717-s001.zip › biology-3660730-supplementary.pdf]

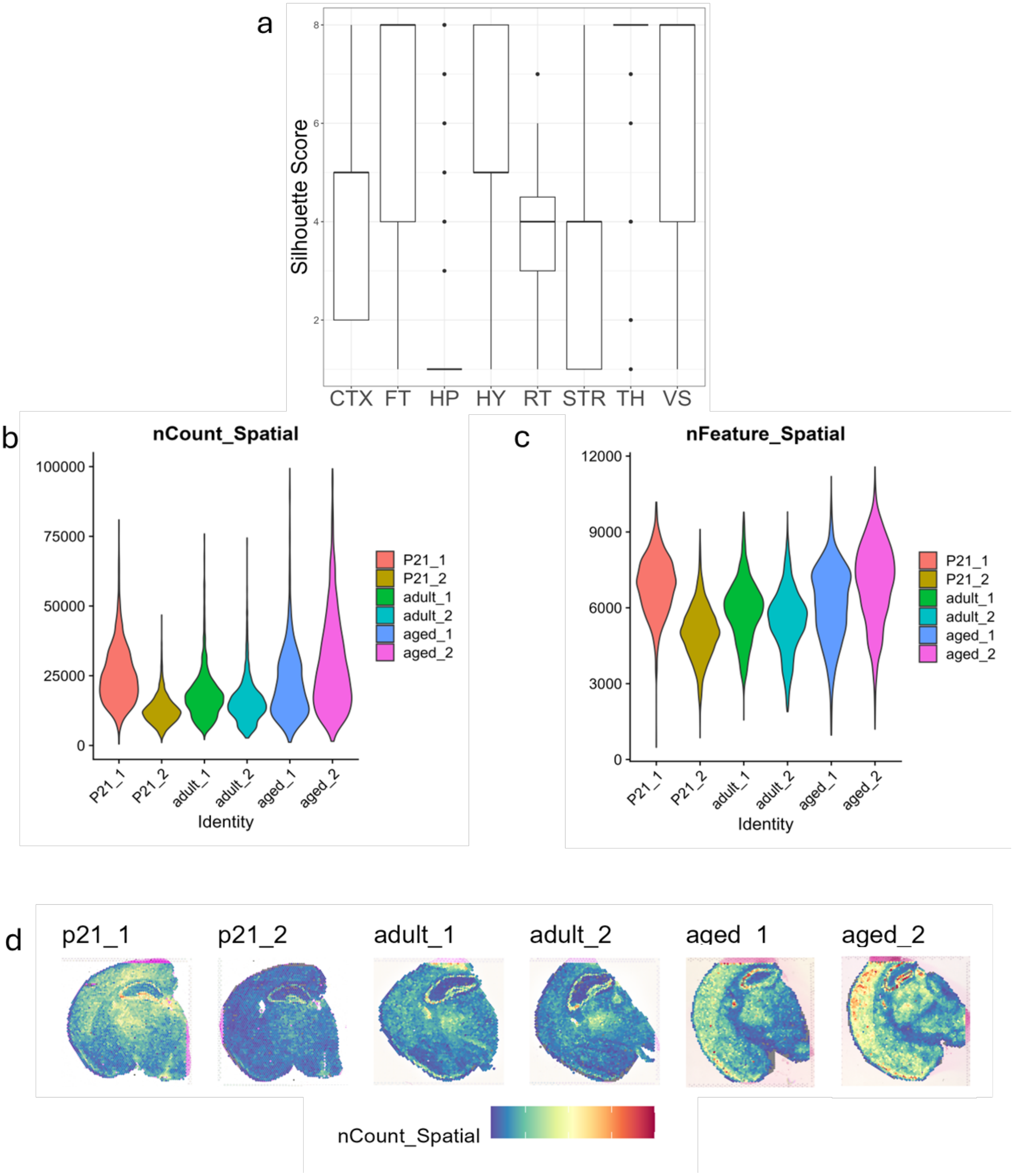

Supplementary Figure S1. Quality Control Metrics and Clustering Robustness of Spatial Transcriptomics Data. a. Silhouette score boxplot showing clustering robustness across brain-region clusters based on the first 10 principal components. Higher silhouette values indicate stronger intra-cluster cohesion and inter-cluster separation. **b.** Violin plot showing the distribution of UMI counts per sample. **c.** Violin plot showing the distribution of detected gene counts per sample. **d.** Spatial density plots of UMI counts across samples.

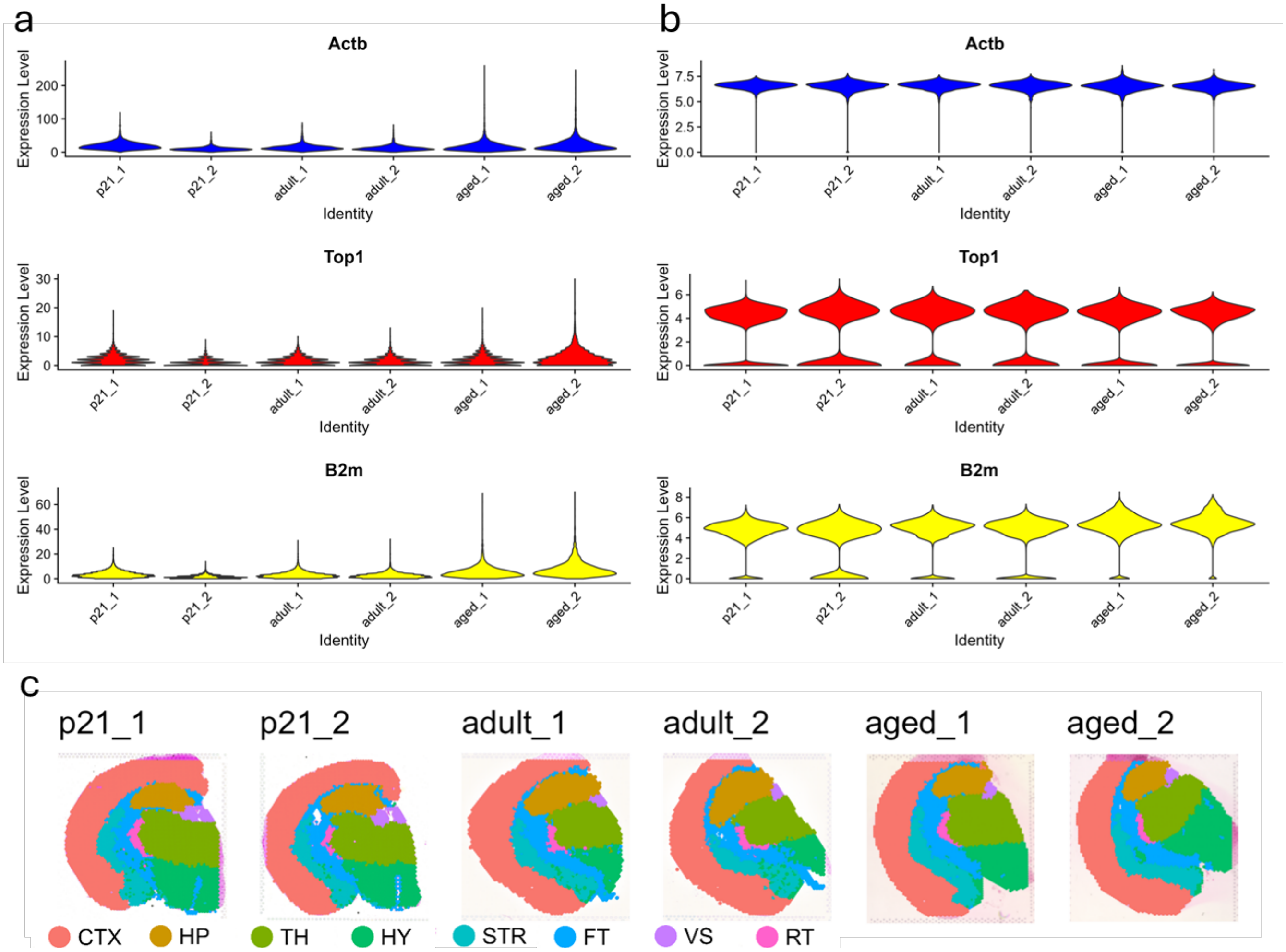

Supplementary Figure S2. Replicate Correlation and Housekeeping Gene Normalization. **a.** Violin plots showing expression levels of housekeeping genes (*Actb*, *Top1*, and *B2m*) across samples before normalization. **b.** Violin plots showing expression of these housekeeping genes after normalization and log transformation. **c.** SpatialDimPlot showing clustering of whole-brain sections, with consistent spatial patterns observed across biological replicates.

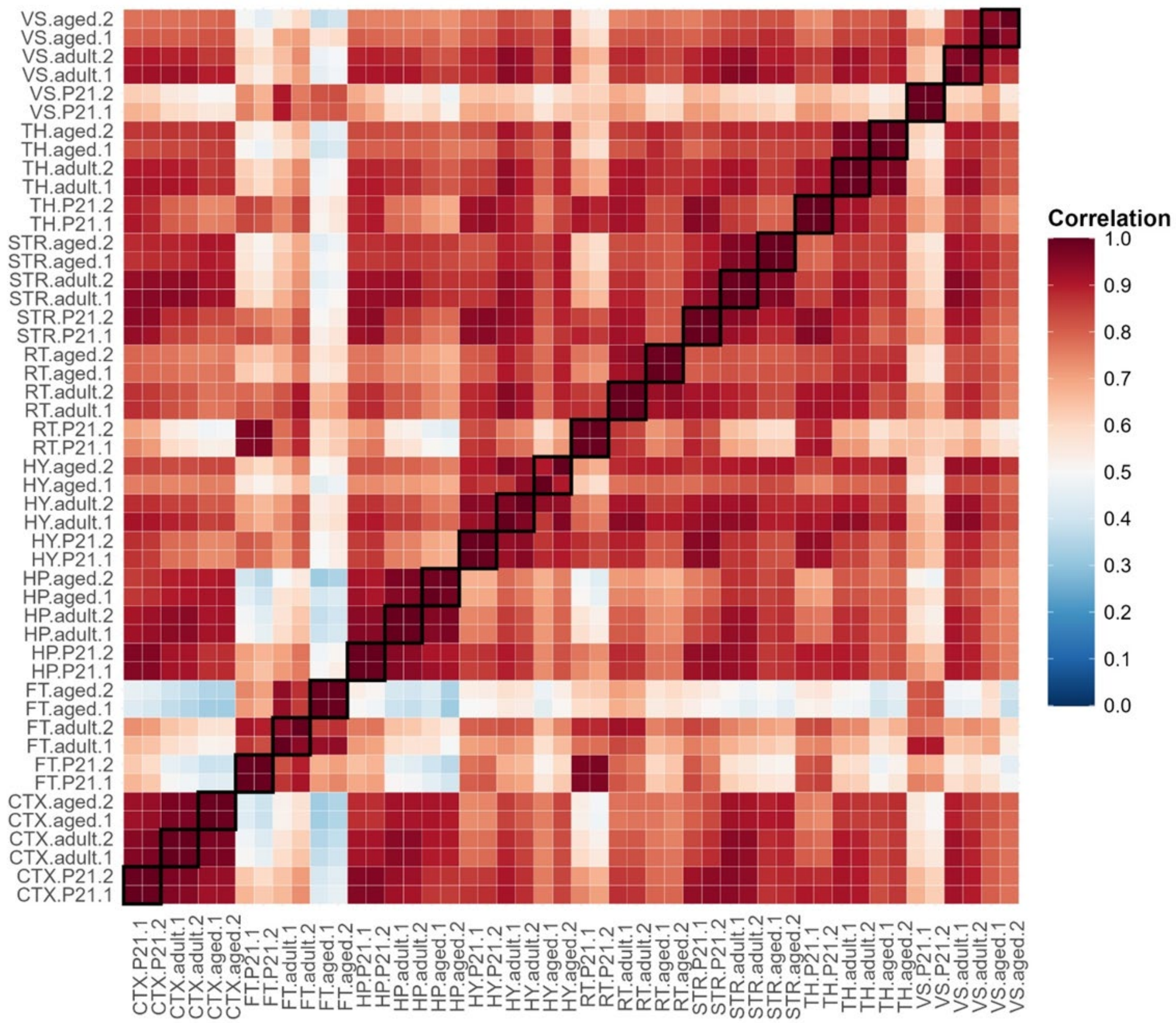

Supplementary Figure S3. Gene Expression Correlation Between Biological Replicates. **a.** Heatmap showing pairwise Pearson correlation of gene expression between biological replicates across brain regions, demonstrating high reproducibility.

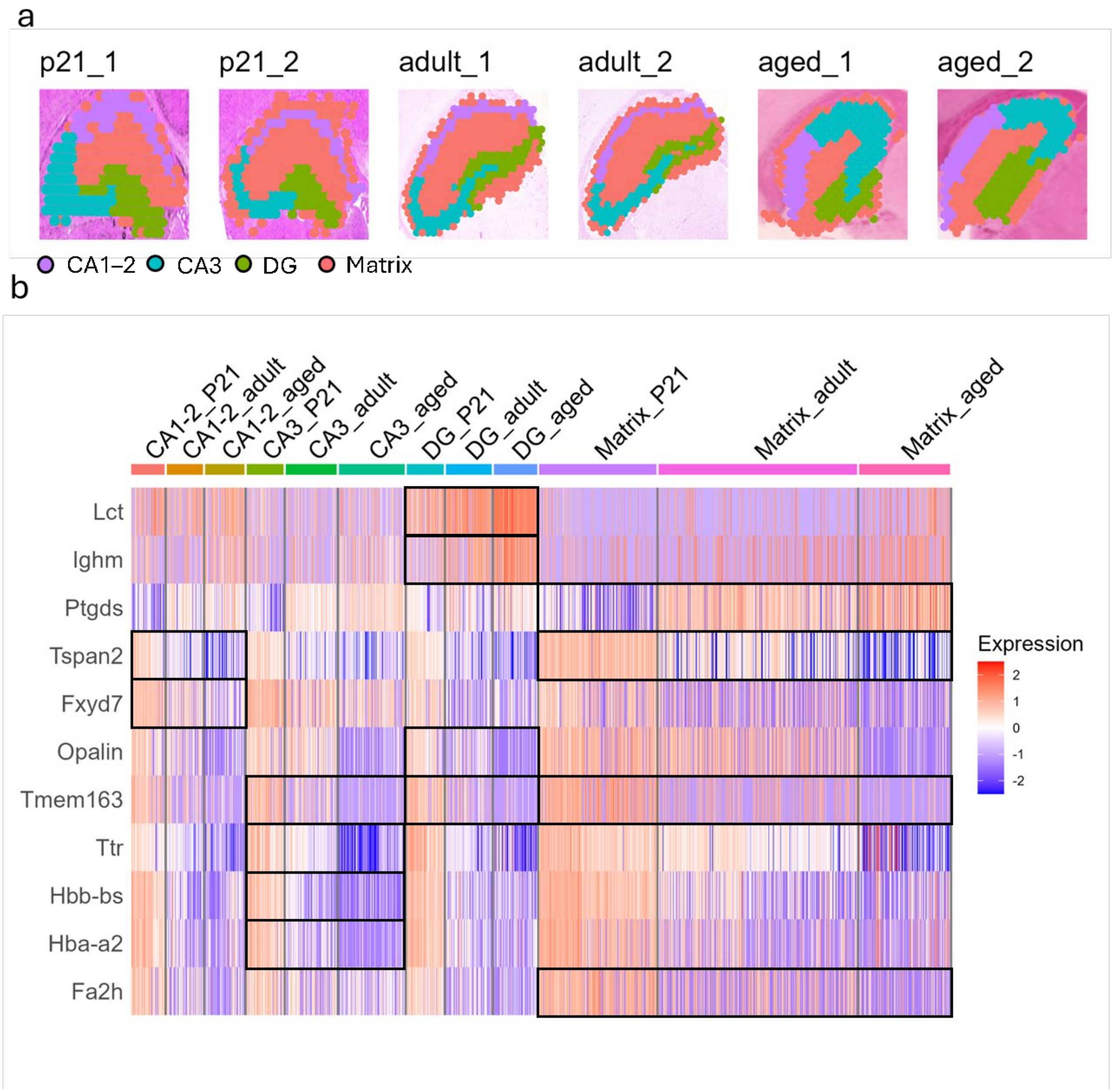

Supplementary Figure S4. Subregion-Specific Spatial Clustering and Differential Gene Expression in the Hippocampus. a. Spatial clustering of the hippocampal subregions CA1-2, CA3, DG, and matrix, demonstrating reproducibility of subregion-specific transcriptional patterns between replicates. **b.** DEGs trending up and trending down by hippocampal subregion, with significant trends highlighted by black boxes.

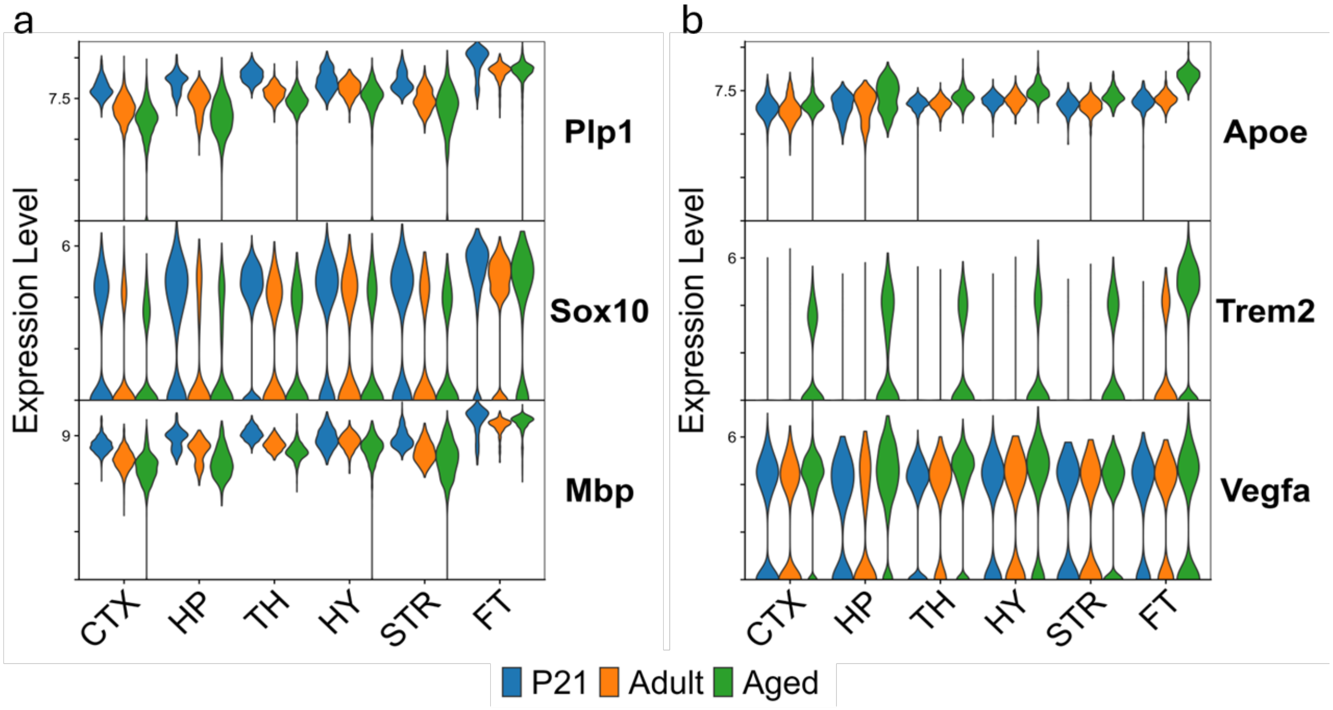

Supplementary Figure S5. Representative Expression Patterns of Myelination- and Inflammation-Associated Genes. a. Violin plots of *Plp1*, *Sox10*, and *Mbp* across hippocampal subregions at each age **b.** Violin plots of *Apoe*, *Trem2*, and *Vegfa*, highlighting spatially variable expression of aging- and inflammation-related genes across subregions.

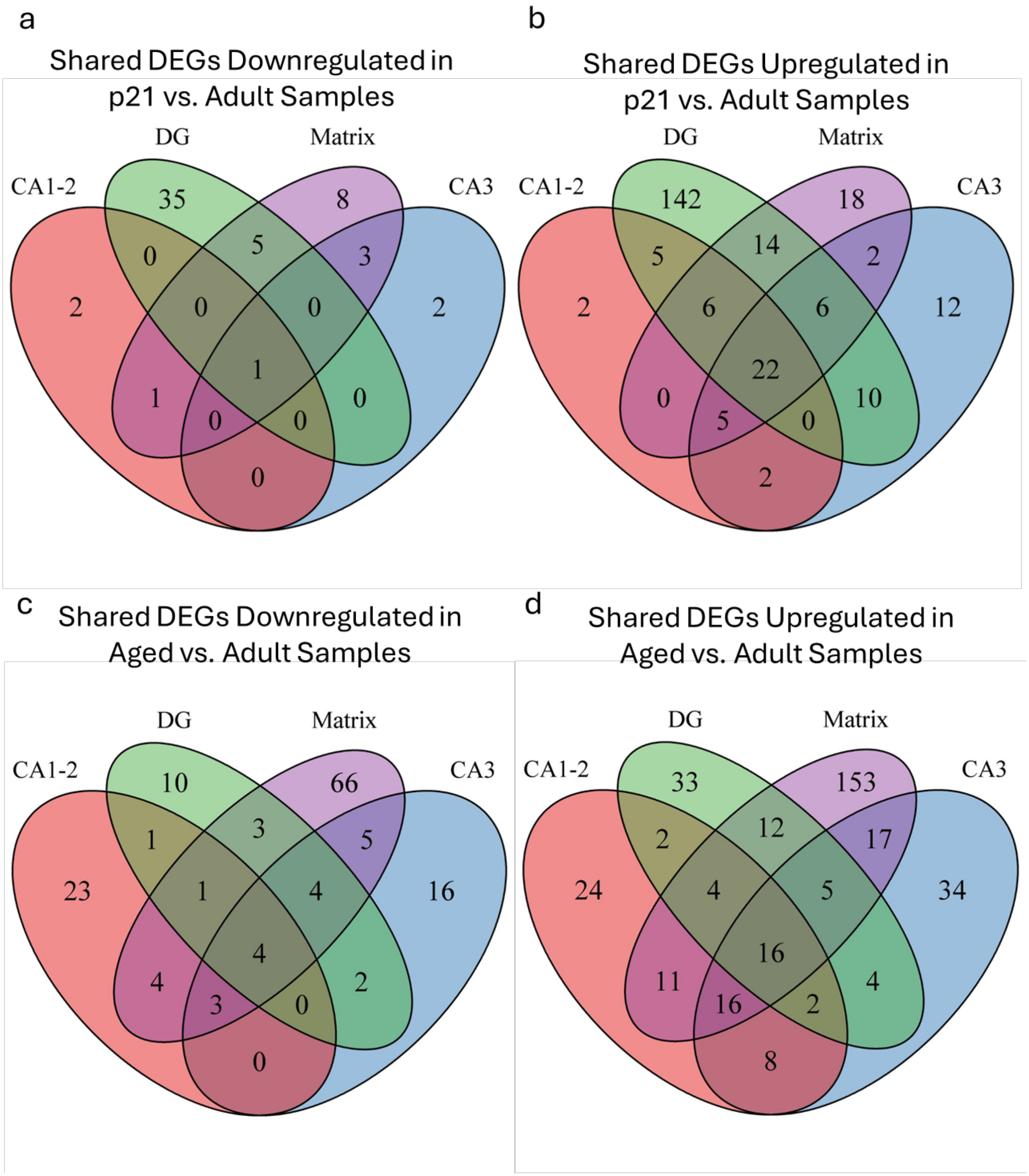

Supplementary Figure S6. Venn Diagrams of DEGs by Sample and Hippocampal Subcluster. a. DEGs downregulated in P21 *vs*. adult samples. **b.** DEGs upregulated in P21 *vs*. adult samples. **c.** DEGs downregulated in aged *vs*. adult samples. **d.** DEGs upregulated in aged *vs*. adult samples.

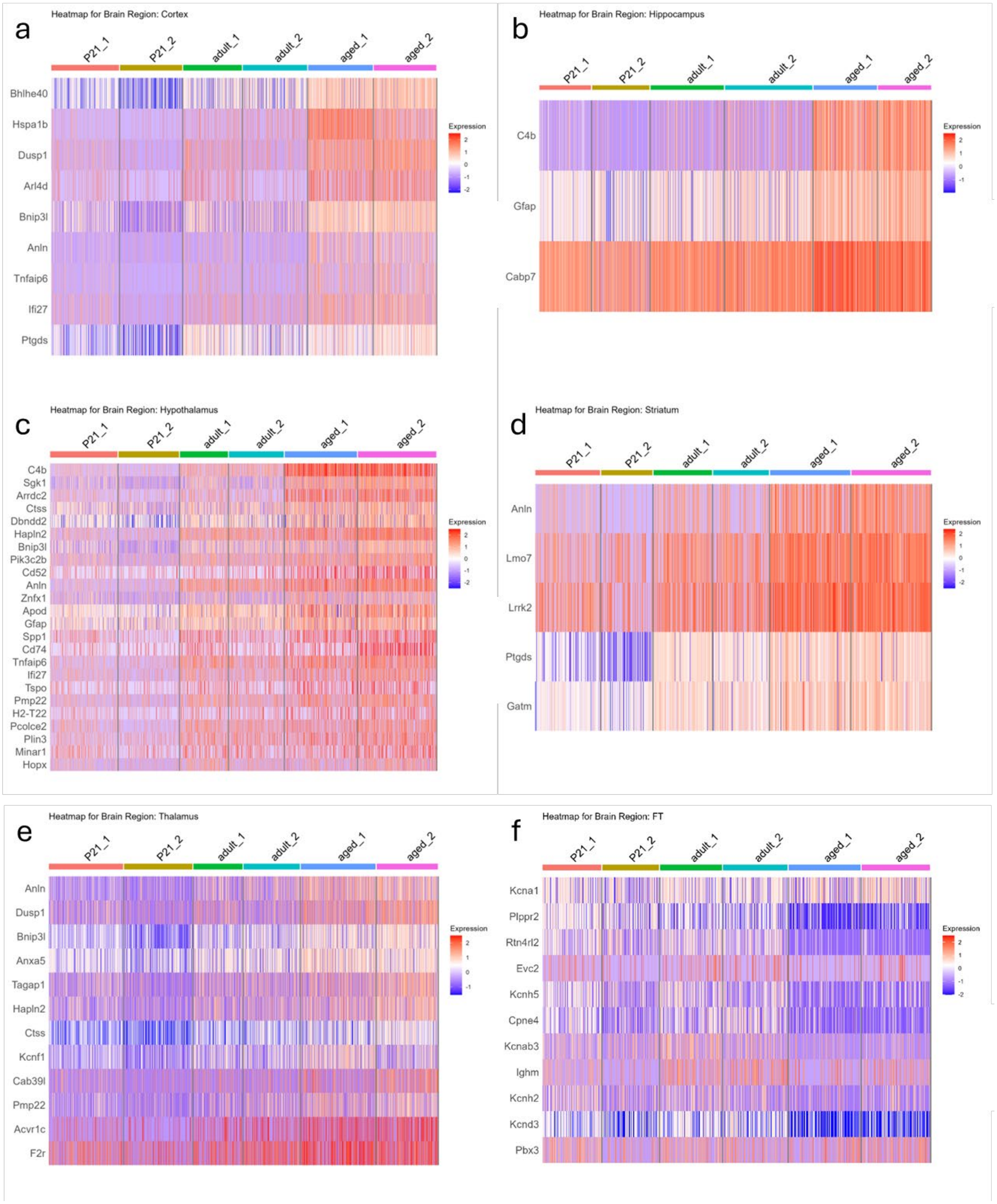

Supplementary Figure S7. Heatmaps of Top Genes Trending Up by Region. **a.** cortex. **b.** hippocampus. **c.** hypothalamus. **d.** striatum. **e.** thalamus. **f.** fiber tracts.

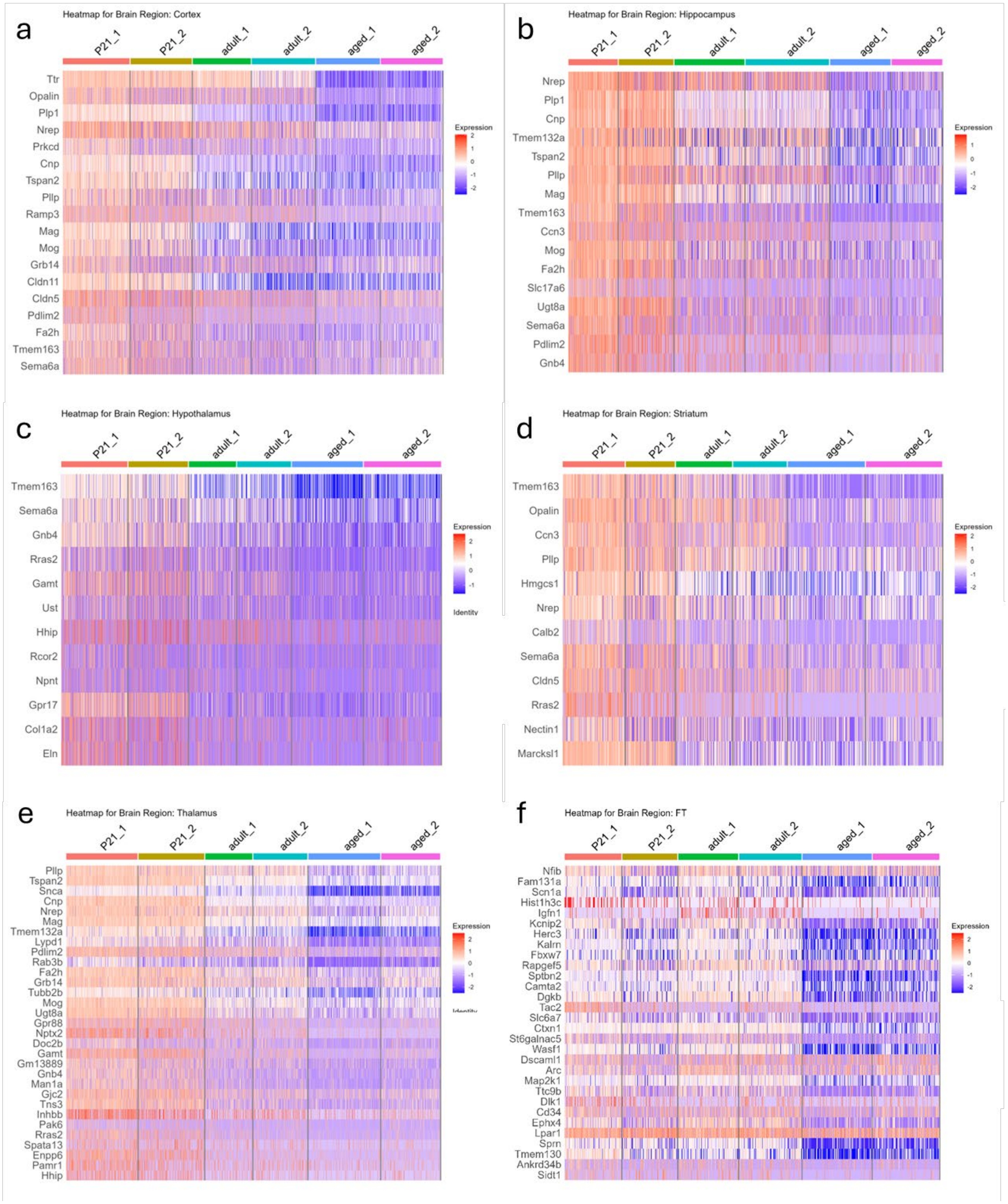

Supplementary Figure S8. Heatmaps of Top Genes Trending Down by Region. **a.** cortex. **b.** hippocampus. **c.** hypothalamus. **d.** striatum. **e.** thalamus. **f.** fiber tracts.

Table S1. Spatial Transcriptomic Spot Counts per Sample and Hippocampal Subregion.

Sample	Subregion	Spot_Count
P21_1	CA1-2	46
P21_1	CA3	53
P21_1	DG	47
P21_1	Matrix	96
P21_2	CA1-2	30
P21_2	CA3	31
P21_2	DG	37
P21_2	Matrix	171
adult_1	CA1-2	39
adult_1	CA3	51
adult_1	DG	54
adult_1	Matrix	200
adult_2	CA1-2	43
adult_2	CA3	65
adult_2	DG	50
adult_2	Matrix	252
aged_1	CA1-2	44
aged_1	CA3	95
aged_1	DG	45
aged_1	Matrix	113
aged_2	CA1-2	46
aged_2	CA3	55
aged_2	DG	54
aged_2	Matrix	94

Supplementary Table S2. Genes Associated with Enriched GO Terms for P21 DEGs

Description	region	direction	pvalue	p.adjust	geneID
regulation of neurogenesis	CTX	Up	1.41E-16	7.45E-14	Mag/Mbp/Sirt2/Ctnnb1/Ncan/Sox10/Sema6a/Gjc2/Dpysl5/Fn1/Kdr/Sox8/Myrf/Islr2/Hapln1/Cers2/Cd24a/Met/Plxnb3/Vcan/Nrp1/Itgb1/Tnfrsf12a/Fstl4/Nkx6-2/Nkx2-2/Sema3f/Ngf
regulation of neurogenesis	HP	Up	1.47E-16	2.53E-13	Mag/Sirt2/Mbp/Marcks/Sema6a/Sox10/Gjc2/Ncan/Dpysl5/Enpp2/Sox8/Dcx/Islr2/Cd24a/Rnd2/Mycn/Fn1/Sox11/Ache/Vcan/Nkx6-2/Nrp1/Sema4g/Plxnb3/Kdr/Oprm1/Nog/Nkx2-2/Sema3c/Plxna3/Ldlr/Sema5b
regulation of neurogenesis	TH	Up	5.37E-12	9.70E-09	Sirt2/Mbp/Mag/Marcks/Ncan/Sox10/Oprm1/Dpysl5/Ache/Hap1/Gjc2/Sox8/Tnr/Cdh4/Ptprf/Itgb1/Cers2/Fn1/Ldlr/Sema4d/Plxnb3/Kdr/Adcyap1/Vcan/Shh/Igf1/Smurf1/Nog/Hapln1
regulation of neurogenesis	HY	Up	1.55E-07	0.000124303	Sirt2/Marcks/Hap1/Ache/Dpysl5/Otp/Sox10/Sema5a/Mag/Sema6a/Sox8/Cers2/Heyl/Efna5/Bdnf/Chodl/Trpc5/Cd24a/Nrp1
regulation of neurogenesis	HY	Down	1.69E-06	0.001113713	Il33/Trf/Hapln2/Appl2/Sema7a/Hapln4/Gfap/Itpka/Shtn1/Hltf/Epha7/Daam2/Pax6/Aspa/Tgm2/Lhx2/Sgk1
regulation of neurogenesis	STR	Up	1.38E-07	2.33E-05	Sirt2/Mbp/Mag/Sox10/Sema6a/Ncan/Gjc2/Fn1/Sox8/Itgb1/Cxcl12/Kdr/Myrf/Cers2/Id1
regulation of neurogenesis	FT	Up	0.000547213	0.025271917	Sema4a/Ndel1/Kalrn/Epha7/Dbn1/Gh/Dpysl5/Sema3c/Vcan/Map2k1/Dnajb11/Rest/Wdr62/Sema3d/Ptprf/Bdnf/Idh2
gliogenesis	CTX	Up	4.10E-20	1.30E-16	Mag/Tspan2/Mal/Cnp/Plp1/Sirt2/Fa2h/Ctnnb1/Ncan/Sox10/Olig1/Agt/Gjc2/Fn1/Sox8/Cd9/Opalin/Apcdd1/Dusp15/Myrf/Rras2/Sox4/Hapln1/Cers2/Erbb3/Vcan/Pou3f1/Nkx6-2/Nkx2-2
gliogenesis	HP	Up	2.09E-17	7.19E-14	Plp1/Tuba1a/Mag/Cnp/Mal/Tspan2/Sirt2/Fa2h/Sox10/Gjc2/Cdk5r2/Ncan/Agt/Enpp2/Sox8/Sox4/Olig1/Dusp15/Dcx/Mycn/Erbb3/Fn1/Sox11/Vcan/Cntnap2/Nkx6-2/Rras2/Nog/Nkx2-2/Ldlr
gliogenesis	TH	Up	1.53E-13	5.54E-10	Plp1/Cnp/Tspan2/Mal/Sirt2/Mag/Tuba1a/Fa2h/Ncan/Sox10/Olig1/Gjc2/Sox8/Rras2/Dusp15/Cd9/Cers2/Fn1/Ldlr/Erbb3/Apcdd1/Sox4/Adcyap1/Vcan/Shh/Igf1/Nog/Hapln1
gliogenesis	TH	Down	0.000565064	0.041072951	Il33/Hapln4/Nr1d1/Nr3c1/Lef1/Hapln2/Nfix/Pmp22/Itgb4/Lgi4/Nab2
gliogenesis	HY	Down	1.07E-05	0.002992116	Il33/Trf/Hapln2/Hapln4/Itgb4/Gfap/Ndrg1/Lgi4/Nfix/Daam2/Pmp22/Pax6/Aspa/Myoc
gliogenesis	STR	Up	6.85E-17	4.34E-14	Plp1/Cnp/Tspan2/Sirt2/Mal/Mag/Fa2h/Sox10/Olig1/Agt/Ncan/Gjc2/Dusp15/Fn1/Cd9/Sox8/Opalin/Rras2/Sox4/Erbb3/Apcdd1/Myrf/Cers2
myelination	CTX	Up	3.20E-17	2.53E-14	Mag/Tspan2/Mal/Plp1/Mbp/Sirt2/Ugt8a/Nfasc/Fa2h/Ctnnb1/Gal3st1/Sox10/Tmem163/Pllp/Cldn5/Cd9/Myrf/Erbb3/Pou3f1/Nkx6-2
myelination in peripheral nervous system	CTX	Up	0.001811854	0.018667406	Sirt2/Fa2h/Pou3f1
myelination	HP	Up	1.64E-11	4.71E-09	Plp1/Mag/Mal/Tspan2/Sirt2/Ugt8a/Nfasc/Mbp/Tmem163/Fa2h/Gal3st1/Sox10/Fyn/Pllp/Erbb3/Cntnap2/Nkx6-2
myelination	TH	Up	2.88E-10	6.93E-08	Plp1/Tspan2/Mal/Sirt2/Mbp/Mag/Ugt8a/Nfasc/Fa2h/Sox10/Gal3st1/Fyn/Pllp/Cd9/Erbb3/Igf1/Rxrg
myelination	HY	Up	1.98E-07	0.000127436	Sirt2/Nfasc/Tspan2/Fyn/Tmem163/Plp1/Ugt8a/Mal/Sox10/Gal3st1/Mag/Fa2h
myelination in peripheral nervous system	HY	Down	7.76E-06	0.002641897	Itgb4/Ndrg1/Lgi4/Pmp22/Myoc
myelination	HY	Down	0.000273308	0.017098575	S100b/Trf/Bcas1/Itgb4/Ndrg1/Lgi4/Pmp22/Myoc

Supplementary Table S2. Genes Associated with Enriched GO Terms for P21 DEGs

myelination	STR	Up	3.90E-17	3.29E-14	Plp1/Tspan2/Sirt2/Mal/Mbp/Mag/Ugt8a/Nfasc/Tmem163/Fa2h/Gal3st1/Fyn/Pllp/Sox10/Cd9/Cldn5/Erbb3/Myrf
learning	HP	Up	0.000178101	0.003689247	Tuba1a/Nptx2/Agt/Hmgcr/Gpr88/Grm4/C1ql1/Cntnap2/Nog
learning	TH	Up	2.94E-05	0.001299703	Tuba1a/Hmgcr/Nptx2/Tnr/Gpr88/B4galt2/Itgb1/Nts/Nog/Tac1/Pak6
learning	HY	Down	0.000283096	0.017098575	Brsk1/Meis2/Slc24a2/Ppp1r1b/Synpo/Nfix/Neurod2/Sgk1
learning	STR	Up	0.004639333	0.042715604	Lgmn/Hmgcr/Agt/Nptx2/Itgb1
learning	FT	Up	0.000346489	0.018745078	Slc12a5/Jph3/Kmt2a/Kalrn/Grm4/Clstn3/Agt/Ube3a/Adgrb3/Bdnf
postsynaptic density organization	CTX	Up	0.004822496	0.034958449	Itgb1/Cbln1/Zdhhc12
postsynaptic density organization	FT	Down	0.000111387	0.032281836	C1ql2/Zdhhc12/Adgrl3/Nptx1/Itgb3
reproductive behavior	TH	Down	0.000233205	0.031006527	Crebrf/Nr3c1/Ar/Zfx/Thrb
reproductive behavior	STR	Down	1.10E-05	0.016741031	Crebrf/Zfx/Thrb/Ar
regulation of chemokine production	HP	Down	0.000114993	0.035113456	Il33/Socs5/Erbin/Egr1
circadian rhythm	CTX	Down	5.40E-05	0.035154594	Ptgds/Bhlhe40/Egr1/Nr1d1/Nr1d2/Bhlhe41/Nudt12
circadian rhythm	HP	Down	0.000131449	0.035113456	Ptgds/Bhlhe40/Nr1d2/Egr1/Kmt2a
circadian rhythm	TH	Down	0.00071247	0.046397809	Ptgds/Bhlhe40/Adcy1/Egr1/Nr1d1/Btbd9/Nudt12/Srebf1
regulation of neurogenesis	DG	Up	7.31E-15	5.07E-12	Mbp/Mag/Enpp2/Dpysl5/Sirt2/L1cam/Dcx/Sema3c/Sox11/Cd24a/Rnd2/Sema4g/Sox10/Mycn/Robo1/Gjc2/Nrp1/Sema6a/Nkx6-2/Dll3/Myrf/Sema6c/Cxcr4/Oprm1/Adcyap1
regulation of neurogenesis	CA12	Up	0.000451309	0.011159652	Mbp/Mag/Sirt2/Sox8/Sox10
regulation of neurogenesis	CA3	Up	3.60E-06	0.000293409	Mag/Mbp/Sox10/Gjc2/Sema6a/Plxnd1/Sox8/Sox11
regulation of neurogenesis	Matrix	Up	2.08E-08	2.66E-06	Mbp/Mag/Sirt2/Marcks/Sema6a/Gjc2/Sox10/Sox8/Dpysl5/Mycn/Dcx
myelination	DG	Up	3.27E-13	1.01E-10	Plp1/Mbp/Mal/Tspan2/Mag/Ugt8a/Sirt2/Fa2h/Tmem163/Cd9/Gal3st1/Sox10/Pllp/Nkx6-2/Myrf/Cxcr4
myelination	CA12	Up	1.15E-17	4.15E-15	Plp1/Mal/Mbp/Mag/Sirt2/Tspan2/Fa2h/Ugt8a/Pllp/Tmem163/Gal3st1/Sox10
myelination	CA3	Up	2.34E-12	8.89E-10	Plp1/Mag/Mal/Tspan2/Ugt8a/Mbp/Tmem163/Fa2h/Sox10/Gal3st1
myelination	Matrix	Up	6.96E-16	3.26E-13	Plp1/Mbp/Mal/Mag/Tspan2/Sirt2/Ugt8a/Tmem163/Fa2h/Gal3st1/Sox10/Erbb3/Cntnap2
gliogenesis	DG	Up	3.33E-19	9.24E-16	Plp1/Mal/Tuba1a/Tspan2/Cnp/Mag/Enpp2/Sirt2/Dcx/Fa2h/Sox11/Cd9/Dusp15/Sox10/Mycn/Olig1/Gap43/Sox4/Dner/Gjc2/Agt/Opalin/Nkx6-2/Dll3/Myrf/Cxcr4/Adcyap1
gliogenesis	CA12	Up	7.54E-14	1.59E-11	Plp1/Mal/Cnp/Mag/Sirt2/Tspan2/Fa2h/Rras2/Sox8/Sox10/Agt/Olig1
gliogenesis	CA3	Up	1.62E-10	3.08E-08	Plp1/Cnp/Mag/Mal/Tspan2/Fa2h/Sox10/Gjc2/Sox4/Sox8/Sox11
gliogenesis	Matrix	Up	4.28E-13	1.20E-10	Plp1/Cnp/Mal/Mag/Tspan2/Sirt2/Fa2h/Gjc2/Sox10/Sox8/Erbb3/Cntnap2/Mycn/Dcx
ensheathment of neurons	DG	Up	2.75E-14	1.09E-11	Plp1/Mbp/Mal/Cldn11/Tspan2/Mag/Ugt8a/Sirt2/Fa2h/Tmem163/Cd9/Gal3st1/Sox10/Pllp/Nkx6-2/Myrf/Cxcr4
ensheathment of neurons	CA12	Up	1.76E-19	9.55E-17	Plp1/Mal/Mbp/Cldn11/Mag/Sirt2/Tspan2/Fa2h/Ugt8a/Pllp/Tmem163/Gal3st1/Sox10
ensheathment of neurons	CA3	Up	7.00E-14	3.99E-11	Cldn11/Plp1/Mag/Mal/Tspan2/Ugt8a/Mbp/Tmem163/Fa2h/Sox10/Gal3st1
ensheathment of neurons	Matrix	Up	2.11E-17	1.49E-14	Plp1/Cldn11/Mbp/Mal/Mag/Tspan2/Sirt2/Ugt8a/Tmem163/Fa2h/Gal3st1/Sox10/Erbb3/Cntnap2

Supplementary Table S3. Genes Associated with Enriched GO Terms for Aged DEGs

Description	region	direction	pvalue	p.adjust	geneID
immune response-activating signaling pathway	CTX	Up	5.995E-05	1.58E-03	Prkcb/Crkl/Fosl2/Nr4a3/Gramd4/Cacnb3/Arrb2/Rtn4/Eif2b4/Otud4/Slc15a4/Mapkapk2/Nek7/Ecsit/Sarm1/Slc39a6/Zdhhc5/Wdfy1/Bcl2/Nfkbiz/Tarbp2/Blnk/Irgm1
immune response-activating signaling pathway	HP	Up	2.062E-06	2.18E-04	Fcer1g/Arrb2/Pdpk1/Eif2b4/Nek7/Laptm5/Trem2/Mfhas1/Nr4a3/Crkl/Cacnb3/Blnk/Cyba/Sarm1/Gramd4/Irgm1/Fosl2/Rela/Tifa/Zdhhc5/Nfkbia/Tarbp2/Usp12/Mapkapk2/Unc93b1/Irf1/Zc3hav1/Ezr
immune response-activating signaling pathway	TH	Up	6.923E-06	3.81E-04	Rtn4/Usp9x/Crkl/Nek7/Pdpk1/Slc15a4/Otud4/Laptm5/Phb2/Arrb2/Eif2b4/Nfkbia/Fcer1g/Trem2/Ecsit/Wdfy1/Cmtm3/Mfhas1/Irgm1/Mapkapk2/Sarm1/Gramd4/Tyrobp/Cyba/Tarbp2/Nfam1
immune response-activating signaling pathway	HY	Up	4.694E-03	3.68E-02	Crkl/Rtn4/Arrb2/Nek7/Gramd4/Laptm5/Eif2b4/Slc15a4/Otud4/Eif2b5/Trem2/Cmtm3/Rela/Wdfy1/Slc39a6/Sarm1/Fcer1g/Sh2b2
immune response-activating signaling pathway	STR	Up	1.281E-04	3.15E-03	Gramd4/Pdpk1/Crkl/Otud4/Slc15a4/Phb2/Eif2b4/Arrb2/Laptm5/Wdfy1/Nr4a3/Ecsit/Fosl2/Fcer1g/Prkcb/Nek7/Trem2/Sarm1/Smpdl3b/Prkch/Tarbp2/Slc39a6/Nckap1l/Cyba/Bcl10/Ripk2
immune response-activating signaling pathway	FT	Up	3.418E-03	4.20E-02	Mef2c/Prkcb/Crkl/Plekha1/Mog/Ighm/Cmklr1/Pum1/Slc15a4/Fcer1g/Mapkapk2/Eif2b1/Fbxl2/Fyn/Nploc4/Lrrc14/Stoml2/Irf3/Btk/Mark4/Ada/Cblb/Casp4/Unc93b1
immune response-activating signaling pathway	FT	Down	1.379E-02	4.42E-02	Cacnb3/Dgkz/Rtn4/Ptprj/Prnp/Hras/Wnk1/Trem2/Flot1/Erbin/Slc39a10/Laptm5/Khdrbs1/Pten/Prkce/Ecsit/Eif2b4/Phb2/Hspd1/Cyba/Pdpk1/Ptprs/Cd47/Arrb2/Ube2n/Tyro3/Pde4b/Lbp/Ufd1/Nr1d1/Foxp1/Nlrc3/Src/C3ar1/Esr1/Icosl
RNA splicing	CTX	Up	2.725E-08	7.62E-06	Atxn7l3/Ildr2/Hnrnpa2b1/Celf1/Srsf2/Prpf19/Strap/Zranb2/Zfp638/Ik/Slc38a2/Hnrnpu/Zpr1/Prpf3/Magohb/Ncbp1/Sf3b4/Rbm17/Srsf1/Luc7l/Snrnp48/Cdc5l/Prpf38b/Lsm3/Rbm12b1/Srrm1/Snrpa/Rbm15b/Ppil1/Lsm2
RNA splicing	HP	Up	9.926E-04	1.19E-02	Atxn7l3/Strap/Slc38a2/Srsf2/Zranb2/Hnrnpa2b1/Zpr1/Rbm17/Sf3b4/Magohb/Rbm15b/Lsm3/Prpf3/Snrpa/Ncbp1/Rbm12b1/Prdx6/Phf5a/Ppil1/Usp49/Snrpb/Ptbp1
RNA splicing	TH	Up	1.986E-04	3.52E-03	Atxn7l3/Ildr2/Prpf19/Strap/Slc38a2/Zranb2/Hnrnpa2b1/Srsf2/Ik/Zfp638/Srsf1/Aqr/Luc7l/Zpr1/Srrm1/Magohb/Rbm17/Snrpa/Kat2b/Ppil2/Sf3b4/Prpf3/Pus7
RNA splicing	HY	Up	5.253E-09	3.16E-06	Zranb2/Prpf19/Hnrnpa2b1/Srsf2/Ik/Atxn7l3/Strap/Srsf1/Bcas2/Slc38a2/Srrm1/Zfp638/Hnrnpu/Prmt1/Cdc5l/Magohb/Luc7l/Sart3/Rbm17/Wdr77/Snrnp48/Aqr/Lsm3/Prpf38b/Zpr1/U2af1/Sf3b4/Rbm15b/Prkrip1/Snrpa/Thoc3
RNA splicing	STR	Up	9.546E-11	6.97E-08	Hnrnpa2b1/Celf1/Strap/Atxn7l3/Zfp638/Srsf2/Prpf19/Rbfox2/Zranb2/Srsf1/Mbnl1/Ik/Aqr/Bcas2/Prmt1/Magohb/Ncbp1/Hnrnpu/Sf3b4/Zpr1/Wbp11/Sart3/Luc7l/Ppil1/Prpf3/Rbm17/Sfswap/Rbm15b/Snrnp27/U2af1/Thoc3/Phf5a/Lsm3/Ncbp2/Kat2b/Snrpa/Rbm12b1/Dhx16/Usp49
RNA splicing	FT	Down	4.128E-03	1.71E-02	Celf4/Celf5/Zmat2/Grsf1/Celf2/Zranb2/Slc38a2/Srsf9/Prpf19/Cirbp/Prdx6/Rbm20/Hnrnpa2b1/Pcbp4/Rbm24/Khdrbs1/Mbnl1/Ik/Thoc7/Srrm1/Tia1/Puf60/Hnrnpa3/Srrm2/Rbm17/Son/Rbm28/Srsf1/Sf3b1/Ivns1abp/Snrpd3/Hnrnpl/Tsen34/Snrnp70/Slirp/Srsf12/Cwc22/Virma/Ptbp3
response to peptide hormone	CTX	Up	6.194E-09	2.68E-06	Egr1/Ctnnb1/Prkcb/Vgf/Irs2/Tsc2/Nr4a1/Nr4a3/Insig2/Cry2/Cry1/Crhbp/Lpin1/Nr4a2/Hadha/Eif2b4/Ctsd/Tsc1/Bcar3/Sgk1/Igf1/Cpeb2/Ccnd3/Sik2/Socs7/Tbc1d4/Rb1/Tns2/Igf2
response to peptide hormone	HP	Up	7.420E-08	2.80E-05	Ctnnb1/Serpina3n/Egr1/Camk2a/Tsc2/Ctsd/Insig2/Hadha/Nr4a2/Pdpk1/Eif2b4/Stat3/Slc39a14/Sgk1/Nfe2l2/Klf15/Nr4a3/Jak1/Tbc1d4/Tsc1/Socs7/Rela/Bcar3/Tns2/Irf1/Igf1/Agtrap/Crhbp/Igf2

Supplementary Table S3. Genes Associated with Enriched GO Terms for Aged DEGs

response to peptide hormone	TH	Up	6.274E-09	5.24E-06	Ctnnb1/Myo5a/Ctsd/Sgk1/Insig2/Tsc2/Agt/Egr1/Klf15/Irs2/Pdpk1/Tsc1/Anxa5/Eif2b4/Lpin1/Hadha/Cited1/Tbc1d4/Agtrap/Nfe2l2/Tns2/Cry1/Cpeb2/Rb1/Acvr1c/Bcar3/Kat2b/Grb10/Pld1/Igf1
response to peptide hormone	HY	Up	5.787E-11	1.39E-07	Ctnnb1/Sgk1/Egr1/Ctsd/Serpina3n/Tsc2/Insig2/Anxa5/Klf15/Hadha/Agt/Rac1/Eif2b4/Lpin1/Tsc1/Pck2/Snx5/Irs2/Leprotl1/Grb10/Stat5b/Eif2b5/Bcar3/Cpeb2/Tbc1d4/Vgf/Rela/Tns2/Rb1/Sh2b2/Nfe2l2/Cited1
response to peptide hormone	STR	Up	3.625E-11	3.43E-08	Ctnnb1/Egr1/Tsc2/Irs2/Sgk1/Pdpk1/Insig2/Vgf/Ctsd/Hadha/Eif2b4/Cry2/Rac1/Nr4a3/Klf15/Snx5/Cdo1/Tbc1d4/Cpeb2/Lpin1/Leprotl1/Rb1/Nfe2l2/Prkcb/Socs7/Gpam/Tsc1/Cry1/Pck2/Agtrap/Foxo4/Tns2/Nr4a1/Leprot/Bcar3/Kat2b/Srebf1
response to peptide hormone	FT	Down	8.539E-09	2.41E-07	Ppp3ca/Camk2a/Ctnnb1/Map2k1/Ctsd/Pak1/Pkm/Tbc1d4/Myo5a/Serpina3n/Prkca/Ptprj/Tsc2/Agtrap/Hras/Rock2/Nr4a2/Ptpre/Ptpra/Hadha/Atp2a2/Nos1/Pten/Gnas/Eif2b4/Gpam/Pip4k2c/C2cd5/Pdpk1/Anxa5/Cfl1/Pdk4/Crk/Opa1/Rab10/Nfe2l2/Rap1gds1/Ahcyl1/Uso1/Rab31/Ptk2/Pip4k2b/Snx5/Rbp4/Crhr1/C1qtnf12/Khk/Cacybp/Src/Slc2a8/Ptprf/Slc25a33
mRNA processing	CTX	Up	6.077E-09	2.68E-06	Pabpn1/Adar/Hnrnpa2b1/Celf1/Srsf2/Prpf19/Strap/Zranb2/Ik/Pcbp1/Hnrnpu/Zpr1/Prpf3/Magohb/Ncbp1/Sf3b4/Cpsf1/Rbm17/Srsf1/Luc7l/Kin/Snrnp48/Cdc5l/Prpf38b/Polr2d/Lsm3/Srrm1/Snrpa/Fastkd5/Rbm15b/Ppil1/Papolg/Lsm2
mRNA processing	HP	Up	1.265E-04	3.29E-03	Pabpn1/Strap/Srsf2/Adar/Zranb2/Hnrnpa2b1/Zpr1/Cpsf1/Rbm17/Sf3b4/Magohb/Rbm15b/Lsm3/Prpf3/Snrpa/Ncbp1/Prdx6/Phf5a/Scaf4/Zc3h3/Ppil1/Usp49/Snrpb/Papolg/Ptbp1/Kin
mRNA processing	TH	Up	1.370E-04	2.71E-03	Pabpn1/Prpf19/Adar/Strap/Zranb2/Hnrnpa2b1/Srsf2/Ik/Srsf1/Pcbp1/Aqr/Luc7l/Zpr1/Scaf4/Cpsf1/Srrm1/Magohb/Rbm17/Snrpa/Ppil2/Sf3b4/Kin/Prpf3/Pus7/Papolg
mRNA processing	HY	Up	4.204E-09	3.16E-06	Pabpn1/Adar/Zranb2/Prpf19/Hnrnpa2b1/Srsf2/Ik/Strap/Srsf1/Pcbp1/Bcas2/Srrm1/Hnrnpu/Cdc5l/Magohb/Luc7l/Sart3/Scaf4/Rbm17/Wdr77/Snrnp48/Aqr/Lsm3/Prpf38b/Cpsf1/Zpr1/U2af1/Polr2d/Sf3b4/Rbm15b/Prkrip1/Snrpa/Thoc3
mRNA processing	STR	Up	4.022E-11	3.43E-08	Pabpn1/Hnrnpa2b1/Celf1/Strap/Adar/Srsf2/Prpf19/Rbfox2/Pcbp1/Zranb2/Srsf1/Mbnl1/Ik/Scaf4/Aqr/Bcas2/Magohb/Ncbp1/Hnrnpu/Sf3b4/Cpsf1/Zpr1/Wbp11/Sart3/Luc7l/Ppil1/Prpf3/Rbm17/Sfswap/Rbm15b/Snrnp27/U2af1/Polr2d/Thoc3/Kin/Phf5a/Lsm3/Ncbp2/Snrpa/Dhx16/Fastkd5/Usp49
mRNA processing	FT	Down	9.234E-04	5.09E-03	Aplp1/Celf4/Celf5/Zmat2/Grsf1/Adar/Pabpn1/Celf2/Zranb2/Akap8l/Srsf9/Prpf19/Cirbp/Prdx6/Rbm20/Hnrnpa2b1/Pcbp4/Rbm24/Khdrbs1/Mbnl1/Ik/Thoc7/Srrm1/Tia1/Puf60/Hnrnpa3/Srrm2/Rbm17/Son/Rbm28/Srsf1/Sf3b1/Snrpd3/Hnrnpl/Ahcyl1/Tsen34/Snrnp70/Slirp/Srsf12/Cwc22/Prmt9/Phrf1/Scaf4/Virma/Ptbp3
learning	HP	Down	1.492E-06	2.46E-04	Pak1/Nrxn1/Ptn/Pde1b/Prkar2b/Ppp1r1b/Deaf1/Adra1b/Tpbg/Chst10
learning	TH	Down	9.734E-12	3.37E-09	Ptn/Pde1b/Syngap1/Synpo/Nrxn3/Jph4/Ppp1r1b/Gabra5/Prkar2b/Reln/Neto1/Gpr88/Nptx2/Cacna1e/Nfix/Slc8a2/Crhr1/Chrd/Pak6/Chrna7
learning	HY	Up	2.161E-03	2.31E-02	Pgrmc1/Sgk1/Shank1/Atp8a1/Ap1s2/Agt/Cln8/Tsc1/C1ql1/Th/Eif2ak4
learning	HY	Down	1.401E-09	2.65E-07	Cck/Ptn/Nrxn1/Pde1b/Syngap1/Nrxn3/Kalrn/Jph4/Synpo/Grin2a/Ppp1r1b/Neurod2/Meis2/Gpr88/Shank2/Deaf1/Prkar2b
learning	STR	Down	5.719E-06	1.25E-03	Nrxn1/Ptn/Cck/Pak1/Pde1b/Prkar2b/Tafa2/Deaf1/Neurod2/Adra1b
learning	FT	Up	6.523E-04	1.28E-02	Ndrg4/Sgk1/Cck/Slc8a2/Gpr88/Grin1/Bdnf/B4galt2/Chst10/Eif2ak4/Deaf1/Ckap5/Abl2/Chrnb2/Adgrb3

Supplementary Table S3. Genes Associated with Enriched GO Terms for Aged DEGs

learning	FT	Down	5.834E-16	7.36E-14	Syngap1/Jph4/Ppp1r1b/Synpo/Kalrn/Ptn/Nrxn1/Gabra5/Pde1b/Pak1/Neurod2/Reln/Tpbg/Cacna1e/Neto1/Tuba1a/Jph3/Kit/Clstn3/Gabrb3/Tac1/Cntnap2/Tafa2/Sorcs3/Nrxn2/Grm5/Hrh3/Slc12a5/Arf4/Fgf13/Cln8/Grin2a/Nts/Nfix/Th/Atxn1/Rgs14/Nptn/Rin1/Dgki/Crhr1/Bche/Mapk8ip2/Glp1r
oligodendrocyte differentiation	CTX	Down	1.692E-07	9.44E-05	Opalin/Ptn/Plp1/Cnp/Tspan2/Mag/Il33/Fa2h
oligodendrocyte differentiation	HP	Down	2.332E-08	1.23E-05	Opalin/Ptn/Plp1/Cnp/Tspan2/Tppp/Mag/Hdac11/Tenm4/Fa2h
oligodendrocyte differentiation	TH	Down	2.908E-07	1.82E-05	Opalin/Ptn/Tppp/Tspan2/Enpp2/Cnp/Mag/Fa2h/Nfix/Tmem98/Vtn/Dusp10
oligodendrocyte differentiation	HY	Down	7.585E-06	2.35E-04	Opalin/Ptn/Lingo1/Mag/Tspan2/Enpp2/Fa2h/Zfp365/Tmem98/Pax6
oligodendrocyte differentiation	STR	Down	1.000E-05	1.60E-03	Ptn/Opalin/Tppp/Lingo1/Cntnap1/Tspan2/Tnfrsf21/Prdm8
oligodendrocyte differentiation	FT	Up	3.725E-03	4.41E-02	Opalin/Plp1/Olig1/Nrg1/Abca2/Sox6/Ckap5/Sox9/Eif2b1/Hdac2
oligodendrocyte differentiation	FT	Down	3.253E-09	1.04E-07	Tppp/Ctnnb1/Ptn/Mag/Tspan2/Cnp/Enpp2/Tenm4/Lingo1/Cntnap2/Ptprj/Fa2h/Hdac11/Ptpra/Il34/Pten/Nfix/Nkx2-2/Eif2b4/Gpm6b/Zfp365/Il33/Prdm8/Clu/Notch1/Exoc4
oligodendrocyte development	HP	Down	1.439E-06	2.46E-04	Plp1/Tppp/Mag/Hdac11/Tenm4/Fa2h
oligodendrocyte development	FT	Down	1.136E-05	1.35E-04	Tppp/Mag/Tenm4/Cntnap2/Fa2h/Hdac11/Pten/Nkx2-2/Eif2b4/Gpm6b/Prdm8/Clu
astrocyte differentiation	HP	Down	6.151E-04	1.46E-02	Gap43/Plp1/Tspan2/Mag/Nr3c1
astrocyte differentiation	TH	Down	4.838E-04	7.73E-03	Tspan2/Mag/Nr3c1/Zeb2/Ntrk3/Dab1/Nfix
astrocyte differentiation	HY	Down	1.839E-03	1.82E-02	Mag/Tspan2/Epha4/Zeb2/Dab1/Pax6
astrocyte differentiation	FT	Down	3.737E-08	8.65E-07	Map2k1/Gfap/Mag/Tspan2/C1qa/Dab1/Epha4/Trem2/Bin1/Prpf19/Nr3c1/Zeb2/Nfix/Nkx2-2/Plpp3/Actr3/Nr1d1/Csf1r/Notch1/Cul4b/Lrp1
regulation of T cell proliferation	DG	Up	3.043E-05	6.46E-03	Ctnnb1/Bcl6/Vsir/Pla2g2f/Sdc4/Laptm5
regulation of T cell proliferation	CA12	Up	4.075E-05	1.71E-03	Ctnnb1/Tsc2/Sdc4/Vsir/H2-D1/Laptm5
regulation of T cell proliferation	CA3	Up	1.343E-04	4.27E-03	Ctnnb1/Tsc2/Vsir/Sdc4/H2-D1/Laptm5
regulation of T cell proliferation	Matrix	Up	8.329E-05	1.44E-03	Ctnnb1/Vsir/Sdc4/H2-D1/Laptm5/Tsc2/Cebpb/Igf2/Igf1
positive regulation of inflammatory response	CA12	Up	2.143E-08	9.72E-06	Il16/Fcgr3/Trem2/Cebpa/H2-D1/Ctsc/Alox5ap/Fcer1g
positive regulation of inflammatory response	CA3	Up	2.450E-05	1.87E-03	H2-D1/Alox5ap/Cebpa/Trem2/Fcer1g/Fcgr3

Supplementary Table S3. Genes Associated with Enriched GO Terms for Aged DEGs

positive regulation of inflammatory response	Matrix	Up	9.529E-08	1.05E-05	H2-D1/Fcer1g/Tnfrsf1a/Cebpa/Fcgr3/Nfkbia/Cebpb/Lgals1/Il16/Trem2/Ptgs2
neuron apoptotic process	CA12	Up	6.064E-09	6.29E-06	Ctnnb1/Agap2/Nr4a2/Apoe/Mt1/Vegfa/Nrp1/Trem2/Amigo2/Arrb2/Fgfr3
neuron apoptotic process	CA3	Up	5.168E-06	1.23E-03	Agap2/Ctnnb1/Nr4a2/Nrp1/Mt1/Zpr1/Vegfa/Trem2/Prodh
neuron apoptotic process	Matrix	Up	4.369E-08	5.82E-06	Apoe/Mt1/Agap2/Ctnnb1/Vegfa/Fgfr3/Prodh/Tnfrsf1a/Pcdhgc3/Cebpb/Ucp2/Usp53/Trem2/Arrb2/Nrp1/Nqo1
regulation of neurogenesis	CA12	Up	1.102E-06	1.61E-04	Ctnnb1/Gfap/Trak2/Apoe/Tsc2/Mfn1/Vegfa/Nrp1/Trem2/Fgfr3
regulation of neurogenesis	CA3	Up	1.064E-06	1.08E-03	Ctnnb1/Gfap/Mfn1/Tsc2/Cdkl5/Nrp1/Sox2/Vegfa/Trem2/Hapln2/Yap1
regulation of neurogenesis	Matrix	Up	1.458E-12	5.63E-09	Apoe/Gfap/Ctnnb1/Vim/Vegfa/Mfn1/B2m/Flt1/Sox2/Fgfr3/Daam2/Sgk1/Id1/Ulk1/Tsc2/Yap1/Per2/Wnt7b /Serpinf1/Trem2/Nrp1/Igf1/Tspo/Rgs14
axon regeneration	CA12	Down	3.988E-05	9.96E-03	Gap43/Nrep/Fkbp1b
neurotransmitter secretion	Matrix	Down	7.807E-07	2.12E-04	Pak1/Snca/Lin7b/Sv2b/Doc2a/Stx1a/Unc13c
vesicle-mediated transport in synapse	Matrix	Down	9.506E-08	6.44E-05	Atp6v1g2/Sncb/Prkar1b/Snca/Sv2b/Ap3m2/Doc2a/Stx1a/Unc13c
synaptic vesicle cycle	Matrix	Down	2.408E-08	3.27E-05	Atp6v1g2/Sncb/Prkar1b/Snca/Sv2b/Ap3m2/Doc2a/Stx1a/Unc13c
